# Integrating text mining with network models for successful target identification: *in vitro* validation in MASH-induced liver fibrosis

**DOI:** 10.3389/fphar.2024.1442752

**Published:** 2024-09-27

**Authors:** Jennifer Venhorst, Roeland Hanemaaijer, Remon Dulos, Martien P. M. Caspers, Karin Toet, Joline Attema, Christa de Ruiter, Gino Kalkman, Tanja Rouhani Rankouhi, Jelle C. B. C. de Jong, Lars Verschuren

**Affiliations:** ^1^ Biomedical and Digital Health, The Netherlands Organization for Applied Scientific Research (TNO), Utrecht, Netherlands; ^2^ Department of Metabolic Health Research, The Netherlands Organization for Applied Scientific Research (TNO), Leiden, Netherlands; ^3^ Department of Microbiology and Systems Biology, The Netherlands Organization for Applied Scientific Research (TNO), Leiden, Netherlands

**Keywords:** disease network, target discovery, target validation, metabolic dysfunction-associated steatohepatitis (MASH), liver fibrosis, text mining, drug discovery, systems biology

## Abstract

An *in silico* target discovery pipeline was developed by including a directional and weighted molecular disease network for metabolic dysfunction-associated steatohepatitis (MASH)-induced liver fibrosis. This approach integrates text mining, network biology, and artificial intelligence/machine learning with clinical transcriptome data for optimal translational power. At the mechanistic level, the critical components influencing disease progression were identified from the disease network using *in silico* knockouts. The top-ranked genes were then subjected to a target efficacy analysis, following which the top-5 candidate targets were validated *in vitro*. Three targets, including EP300, were confirmed for their roles in liver fibrosis. EP300 gene-silencing was found to significantly reduce collagen by 37%; compound intervention studies performed in human primary hepatic stellate cells and the hepatic stellate cell line LX-2 showed significant inhibition of collagen to the extent of 81% compared to the TGFβ-stimulated control (1 μM inobrodib in LX-2 cells). The validated *in silico* pipeline presents a unique approach for the identification of human-disease-mechanism-relevant drug targets. The directionality of the network ensures adherence to physiologically relevant signaling cascades, while the inclusion of clinical data boosts its translational power and ensures identification of the most relevant disease pathways. *In silico* knockouts thus provide crucial molecular insights for successful target identification.

## 1 Introduction

Population aging and increasingly unhealthy lifestyles have resulted in an exponential growth in chronic diseases (Ansah and Chiu, 2023). These chronic diseases often have complex comorbidities and pose challenges to not only the society and individuals but also the pharmaceutical and healthcare industries (Palladino et al., 2016; Cabral et al., 2019; Fermini and Bell, 2022). The increasing need for disease-modifying therapies is offset by the high attrition rates of drugs, partly due to the multifactorial etiologies of the prevalent diseases (Sun et al., 2022; March et al., 2021). The primary source of failure of clinical trials is the lack of demonstrated efficacy (Fogel, 2018; Yamaguchi et al., 2021; Hingorani et al., 2019).

The unmet medical needs in the context of chronic diseases as well as late-stage drug failures due to lack of therapeutic benefits have resulted in a paradigm shift in drug development. The notion of “one disease–one target–one drug” has largely been replaced by biological network approaches (Barabási et al., 2011), where a disease is viewed as a perturbed molecular system with intricate dependencies. Disease progression in the mechanistic network occurs via key nodes (proteins) that can cause system-wide disruption upon intervention to rebalance the biological network and achieve treatment of complex diseases. This approach benefits from recent technological and *in silico* advancements by allowing well-constructed networks with physiological relevance (Silverman et al., 2020). In particular, novel disruptive *in silico* technologies (e.g., artificial intelligence, machine learning, and large language models (LLMs)) and the increased scale of wet-lab capabilities (e.g., high-throughput transcriptomics) that produce large datasets of bioactivity data are critical to translational disease networks (Noor et al., 2023; Brown et al., 2018; Ivanisevic and Sewduth, 2023). Furthermore, the importance of unbiased, data-driven decisions in drug development has been illustrated by Pfizer recently, stressing upon the need for a deeper understanding of the biology of disease (Fernando et al., 2022).

**FIGURE 1:**
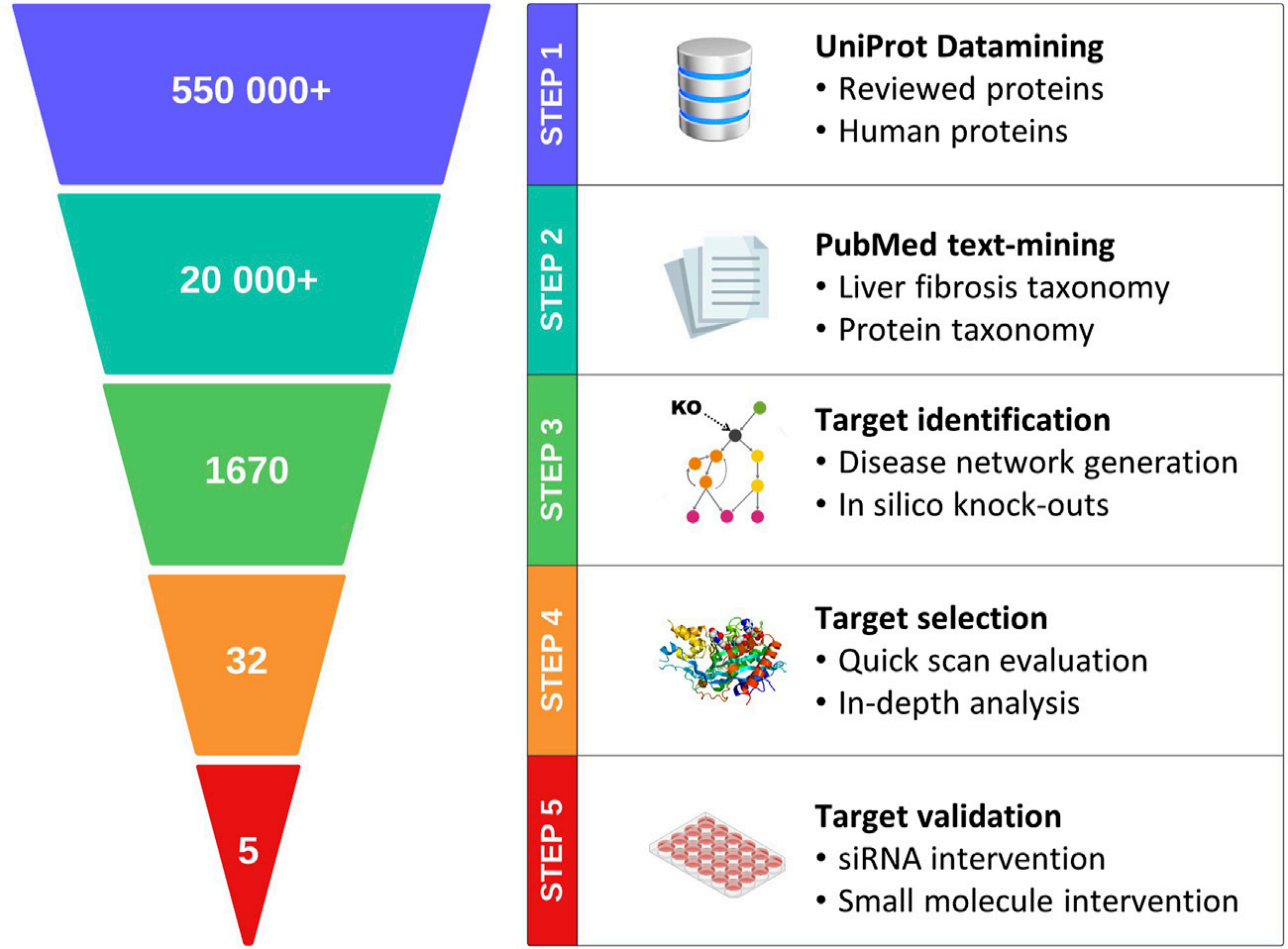
Schematic representation of the major steps in the proposed data-driven pipeline for target discovery.

In this study, we explored the feasibility of incorporating the best practices described above into a generic pipeline for target discovery (Figure 1); this pipeline integrates state-of-the-art technologies with transcriptome data from clinical samples, enabling construction of a contextual, weighted, and directional data-driven disease network. Objective metrics are subsequently used to identify and rank the candidate targets for complex diseases. To validate the proposed approach, metabolic dysfunction-associated steatohepatitis (MASH) and its associated liver fibrosis were selected as a case study. MASH (previously known as non-alcoholic steatohepatitis or NASH) (Rinella et al., 2023) is a severe form of metabolic dysfunction-associated steatotic liver disease (MASLD) that is characterized by steatosis, hepatocellular ballooning, and lobular inflammation in addition to fibrosis (Friedman et al., 2018). A recent study showed that global MASLD rates have increased from 25% to 38% over the past three decades (Younossi et al., 2023); this disease constitutes the main cause of chronic liver disease and is the leading indication for liver transplantation (Zeng et al., 2024; Younossi et al., 2023). Advances have been made toward therapeutics targeting MASH (Harrison et al., 2023), and the pioneer drug resmetirom (Harrison et al., 2024) has only just been approved by the United States Food and Drug Administration (USFDA) earlier this year.

Herein, we propose an approach to construct a weighted and directional liver fibrosis network. To identify the interventional targets, we focused on the disease subnetwork involving growth factor (GF)-related signaling, i.e., using GF receptor (GFR) activation as the molecular initiating event. GFs are critical regulators of wound healing and fibrosis (Seitz and Hellerbrand, 2021). As fibrosis is the most critical predictor of MASH stage and prognosis, collagen deposition was considered the main functional readout for fibrosis in an *in vitro* fibrosis model using either primary hepatic stellate cells (HSCs) or the HSC line LX-2. Using the proposed approach, we identified numerous targets affecting the progression of liver fibrosis. One of these targets is histone acetyltransferase (HAT) EP300, which is used to exemplify the construction, analysis, and validation of the MASH-induced liver fibrosis network for target discovery.

## 2 Materials and methods

### 2.1 *In silico* analyses

#### 2.1.1 Text-mining-based selection of disease network nodes for liver fibrosis

Two taxonomies were used to identify the proteins associated with liver fibrosis. The first is a protein taxonomy as implemented in our in-house target profiling platform TargetTri (www.targettri.com; first accessed on 17/01/2022) (Venhorst and Kalkman, 2024). Briefly, the TargetTri platform contains all human-reviewed proteins deposited in the UniProt database (Bateman et al., 2023); the naming conventions are derived from this database and enriched with additional data sources using UniProt identifiers for cross-mapping of three databases: NCBI (Sayers et al., 2022), ChEMBL (Zdrazil et al., 2023), and HGNC (Seal et al., 2023). As our research group has a lengthy history of performing target assessments both in-house and for third parties (Venhorst et al., 2019), this taxonomy has been extended over the years with manually curated terms to cover knockouts, isoforms, and protein family names, among others. Further enrichment was achieved by targeted efforts to include other sources, such as protein complexes from the complex portal (Meldal et al., 2019).

The second taxonomy describes liver fibrosis at various levels. Here, we relied on our expert knowledge of this metabolic disease as well as recent observations described in literature. The resulting taxonomy includes terms that reflect the cellular (e.g., HSC activation), mechanistic (e.g., extracellular matrix (ECM) cross-linking), and clinical (e.g., enhanced liver fibrosis (ELF) score) aspects of liver fibrosis in subclusters (Supplementary Material S1.1). Subsequently, sentence-level analysis was performed on all PubMed abstracts from the year 2000 onward, which were previously preprocessed, annotated, and stored in the TargetTri platform using our in-house pipeline. This text-mining pipeline downloads PubMed data from the PubMed FTP server (https://ftp.ncbi.nlm.nih.gov/pubmed/updatefiles/) on a daily basis, following which abstracts are tokenized into sentences and then words. Once the word tokens are lemmatized, the two taxonomies are used to automatically annotate the entities. In the preprocessing step, the libraries from the Natural Language Toolkit were used for tokenization of sentences and words, part-of-speech tagging, and lemmatization (Bird et al., 2016). For accurate acronym detection in texts, the algorithm introduced by Mohammed and Nazeer (2013), whose views were based on the work of Schwartz and Hearst (2003) to a great extent, was applied. This algorithm helps identify the acronym–definition pairs in a text based on parameters such as the length of the acronym, candidate words of the definition, presence of parentheses, and specific alphanumeric characters. Both the original data and annotations are stored in ElasticSearch (https://www.elastic.co/) to allow efficient use of the data in the TargetTri platform (Venhorst and Kalkman, 2024).

Sentences containing both a protein and one of the liver fibrosis terms were identified using our taxonomies, as described above. The resulting protein and liver fibrosis occurrences were scored at the publication level per fibrosis cluster. To establish whether the identified proteins themselves represent liver fibrosis hubs, the numbers of neighbors (including known and predicted protein–protein interactions (PPIs) defined using the STRING database and API; https://string-db.org/api; first accessed on 17/01/2022) (Szklarczyk et al., 2021) that were also associated with liver fibrosis based on our text-mining approach were scored. For the STRING search, a significance threshold of a minimum interaction score >0.9 (high confidence) was applied. To assess whether the text mining was able to retrieve genes known to play roles in liver fibrosis, the results were compared against a predictive molecular signature for the onset of MASH-related fibrosis in a translational MASH mouse model (van Koppen et al., 2018).

#### 2.1.2 Construction of a directional, weighted disease network for fibrosis

To construct the liver fibrosis disease network, the top 1,670 ranked proteins (nodes) identified by text mining were used initially. First, these 1,670 proteins were imported into the Ingenuity Pathway Analysis (IPA, Qiagen, United States) platform. Subsequently, the PPIs (edges) and their directionality as defined in the IPA platform were added; this resulted in a directional PPI network containing 999 connected nodes and 671 nodes lacking connections. To connect the latter nodes, STRING (version 11.5) was used to extract PPIs with STRING e-scores (Szklarczyk et al., 2021) equal to or greater than 0.0. The unconnected proteins from IPA that could be connected based on the PPIs defined in STRING were added to the fibrosis disease network. This allowed connection of 665 nodes, resulting in a total network size of 1,664 nodes, and the remaining six nodes were removed. CellTalkDB (Shao et al., 2021) was used to enrich the network with manually selected proteins representing molecular initiating events (6 growth factors and their 12 receptors) and end points (11 ECM proteins) of liver fibrosis (Table 1). The paths between these start and end nodes represent the disease pathways that can be halted or reversed upon therapeutic intervention. The choice of focusing on GF-related signaling for interventional target identification was prompted by the fact that GFs are critical regulators of wound healing and fibrosis (Seitz and Hellerbrand, 2021). In turn, ECM deposition is considered the hallmark of liver fibrosis (Parola and Pinzani, 2024).

**TABLE 1:** Start and end nodes defined for the liver fibrosis disease network. To identify candidate drug targets, all nodes (proteins) in the network were individually knocked out *in silico* while walking over the network from all combinations of start to end nodes using Yen’s K-shortest-path algorithm, as implemented in Neo4J.

Starting nodes	
Ligands	Receptors
CCL2, EGF, FGF21, LEP, PDGFB TGFB1	CCR2, EGFR, LDLR, FGFR1, FGFR2, FGFR3, LEPR, LRP2, PDGFRA, PDGFRB, TGFBR1, and TGFBR2

End nodes	
COL1A1, COL1A2, COL3A1, ELN, FN1, TNC, TIMP1, BGN, FBN1, FBN2, and FBN3	

To assign weights to the edges of the disease network, two data sources were combined: STRING and clinical transcriptomics data from MASH patients. First, the e-scores from STRING were adopted to reflect the confidence on a scale of 0 to 1 for the association between two nodes being true based on experimental evidence (Szklarczyk et al., 2021). The relationships identified by only IPA were assigned a default score of 0.5001. Second, the clinical transcriptome data from liver fibrosis samples (gene expression omnibus (GEO) dataset GSE240729 (Verschuren et al., 2024)) were used for the optimal translational value of the disease network. To calculate the weights of the edges based on transcriptome data, the weights of each of the nodes were calculated by taking the absolute 2logR expression values between the NASH and fibrosis patients (F3/F4) as compared to NASH patients without fibrosis (F0). The weights of the edges were then calculated as the average of the weights of the two connected nodes. Thus, the final weight assigned to each edge in the fibrosis disease network was the sum of the e-score from STRING and weight derived from the clinical transcriptome data. The transcriptome dataset used here is accessible at the NCBI GEO database with accession number GSE240729. The differentially expressed genes (DEGs) across the fibrosis stages were identified using the DESeq2 package in R with *p*-value <0.01, (2logR>0.5 OR 2logR < −1), and avg (nCnts) > 20. For further analysis of the PPI network (Supplementary Material S1.2), the proteins and their respective interactions along with the weights and directionality were imported into Neo4j, a graph database management system (Neo4j, 2016).

#### 2.1.3 *In silico* knockout experiments

*In silico* knockout analyses were performed with Neo4j to examine the behavior of the liver fibrosis disease network after perturbation of specific proteins to identify the candidate drug targets. First, the fibrosis pathways were determined by traversing the disease network from the selected start to end nodes (Table 1); these walks were performed with Yen’s K-shortest-path algorithm (Yen, 1972) available in the Neo4j Graph Data Science Library plugin, where K represents the number of shortest paths that must be computed. The shortest path is the one with the lowest cost, where the cost in this study was based on the total weights of the edges for each of the investigated paths. To analyze the fibrosis pathways, these paths have to represent the shortest paths in the disease network, i.e., paths with the lowest costs and hence total weight. The top-100 shortest paths were used in the analysis. To calculate the cost of each perturbed liver fibrosis path, i.e., start/end node combination, the length and cost of the path determined by Yen’s K-shortest-path algorithm were set as the reference. Subsequently, each node in the disease network was iteratively removed from the network, and the K shortest paths were recalculated and compared with the reference value. The nodes (proteins) for which the costs increased maximally after knockout were selected for further analyses, i.e., the target efficacy assessments.

#### 2.1.4 Target efficacy assessments of the top-ranked target candidates

A two-tiered approach was used to assess whether the top-ranked target candidates were suitable as therapeutic targets for MASH-related liver fibrosis. This analysis is derived from the target assessment workflow described earlier (Venhorst et al., 2019). For the top-30 targets, a quick-scan analysis was performed using database-level information, where the aspects listed in Supplementary Material S1.3 were queried. The top-5 candidate targets were further assessed on the basis of expert analyses of literature. The major criteria included the therapeutic rationale of the target in liver fibrosis, consistency of observations, expression profiles, genetic phenotype, (pre)clinical data, druggability and screening options of the target (*vide infra*), and absence of potential showstopper target-related toxicities. The most promising candidates were then validated in an *in vitro* liver fibrosis model.

### 2.2 *In vitro* analyses

#### 2.2.1 siRNA studies in primary HSCs

The primary HSCs (BioIVT, West Sussex, United Kingdom) were seeded on fibronectin-coated (Roche, Woerden, Netherlands) 24-well culture plates and maintained overnight in a stellate cell medium (STECM) supplemented with 2% (v/v) fetal bovine serum (FBS), 1% (v/v) antibiotic solution, and 1% (v/v) stellate cell growth supplement (all HSC seeding medium materials were from ScienCell, Carlsbad, CA, United States). The HSCs were incubated for 3 days in Accell siRNA Delivery Medium (Dharmacon, Horizon Discovery Ltd.) with or without TGF-ß1 (2 ng/mL; rh-TGFB1 R&D Systems, Minneapolis, MN, United States) and with or without TGF-β1 cotreated with 1 µM siRNAs against EP300 (Accell EP300, Accell eGFP was used as a control). After 3 days, the culture medium was replaced with STECM supplemented with 1% (v/v) FBS, 1% (v/v) antibiotic solution, 1% insulin–transferrin–selenium (ITS), 173 μM of L-ascorbic acid-2-phosphate, 2.5 mM of proline, 2.5 mM of lysine, and 2.5 mM of glycine before culturing for another 4 days. The conditioned culture medium was collected. The cell/matrix fraction was hydrolyzed in 6 M hydrochloric acid and used to determine the collagen protein concentration based on hydroxyproline residues (QZBtiscol, Quickzyme Biosciences, Leiden, Netherlands) and total cell/matrix protein concentration (QZBtotprot, Quickzyme) following the manufacturer’s instructions. The total protein levels were used to correct the collagen concentration per sample.

#### 2.2.2 Compound intervention studies in primary HSCs

The primary HSCs were seeded on fibronectin-coated 24-well culture plates and maintained overnight in STECM supplemented with 2% (v/v) FBS, 1% (v/v) antibiotic solution, and 1% (v/v) stellate cell growth supplement. The HSCs were incubated for 4 days in STECM supplemented with 1% (v/v) FBS and 1% (v/v) antibiotic solution with or without TGF-β1 (2 ng/mL) and TGF-β1 cotreated with the TGFβ type I receptor kinase (ALK5) inhibitor LY-364947 or one of the EP300 inhibitors L002 or inobrodib (CCS1477) (all from MedChemExpress). The cell/matrix fraction was hydrolyzed in 6 M hydrochloric acid and used to determine the collagen protein concentration based on hydroxyproline residues and total cell/matrix protein concentration following the manufacturer’s instructions.

#### 2.2.3 Compound intervention studies in LX-2 cells

Human HSCs (LX-2; Merck; lot 2492302) were seeded on fibronectin-coated 24-well culture plates and maintained in Dulbecco’s modified Eagle’s medium (DMEM) supplemented with 2% (v/v) FBS and 1% (v/v) antibiotic solution. The cells were incubated for 7 days in DMEM supplemented with 1% (v/v) FBS, 1% (v/v) antibiotic solution, 1% ITS, 173 μM of L-ascorbic acid-2-phosphate, 2.5 mM of proline, 2.5 mM of lysine, and 2.5 mM of glycine with or without TGF-ß1 (5 ng/mL) in the absence or presence of target specific compounds as well as medium change after 4 days. The conditioned culture media were collected after 4 and 7 days. After 7 days of culturing, the cell/matrix fraction was washed with phosphate-buffered saline (PBS) and hydrolyzed in 6 M hydrochloric acid before being used to determine the collagen protein concentration based on hydroxyproline residues and total cell/matrix protein concentration following the manufacturer’s instructions. The total protein levels were used to correct the collagen concentration per sample. The following inhibitory compounds were used (all at a final concentration of 0.22% dimethyl sulfoxide (DMSO)) for EP300: inobrodib (1–10 µM) and L002 (1–10 µM).

### 2.3 Statistical analyses

The statistical differences were determined using IBM SPSS Statistics 29 (IBM, NY, United States), and normality was assessed with the Shapiro–Wilk test. If the data were normally distributed, then a one-way ANOVA followed by Dunnett’s test was used, in which the groups exposed to treatments were compared to the controls. If the data were not normally distributed, then a Kruskal–Wallis test followed by Mann–Whitney U test was used. Two-tailed *p*-values were used, and *p*-values less than 0.05 were considered to be statistically significant.

## 3 Results

The data-driven pipeline for target discovery (Figure 1) starts with the selection of the text-mining-based disease network nodes (proteins) using expert-curated disease–protein taxonomies. The nodes were subsequently translated into a connected, directional, and weighted disease network. *In silico* knockout experiments were then performed to identify the hub genes that play apparent crucial roles in disease progression. Based on a two-tiered target efficacy assessment (quick-scan and in-depth analyses), the proteins were ranked, and the top-ranked proteins were finally validated using functional *in vitro* assays.

### 3.1 MASH/liver fibrosis disease network

Using the fibrosis cluster and fibrotic neighbor scores derived from text mining (Section 2.1.1), a ranked list of proteins involved in liver fibrosis was generated. Of the 20,000+ human-reviewed proteins in UniProt, 7,895 proteins were identified to have associations with at least one of the terms in our disease taxonomy. The subsequent rankings were based on four separate measures capturing the main pathological processes of liver fibrosis to ensure that the proteins that are less investigated but still linked to disease pathogenesis are included. These measures were as follows: 1) publication count in the HSC cluster; 2) hub gene character measured as the number of fibrotic neighbors; 3) sum of the ratios of liver fibrosis to total publications and liver fibrosis to total neighbors; 4) total number of publications associated with a liver fibrosis term. In particular, the HSC cluster was chosen because HSC activation is the key event in hepatic fibrosis. The activation of these quiescent cells into ECM-producing myofibroblasts amounts to excessively produced ECM proteins during liver fibrosis. The focus on HSC processes is also aligned with the *in vitro* models used for target validation. The top-600 ranked proteins from each of the four measures described above were selected for the initial construction of a fibrosis disease network, resulting in 1,670 unique proteins. To ensure that this selection procedure included the “usual suspects” (i.e., genes known to be involved in fibrosis progression), the recall of our previously described fibrosis signature (van Koppen et al., 2018) was assessed. This predictive molecular signature reflects the active fibrotic processes. With a combined recall of 83/90 human-mapped signature genes, the selection process was deemed fit-for-purpose for capturing the relevant fibrotic processes.

### 3.2 *In silico* knockouts for target selection

**FIGURE 2:**
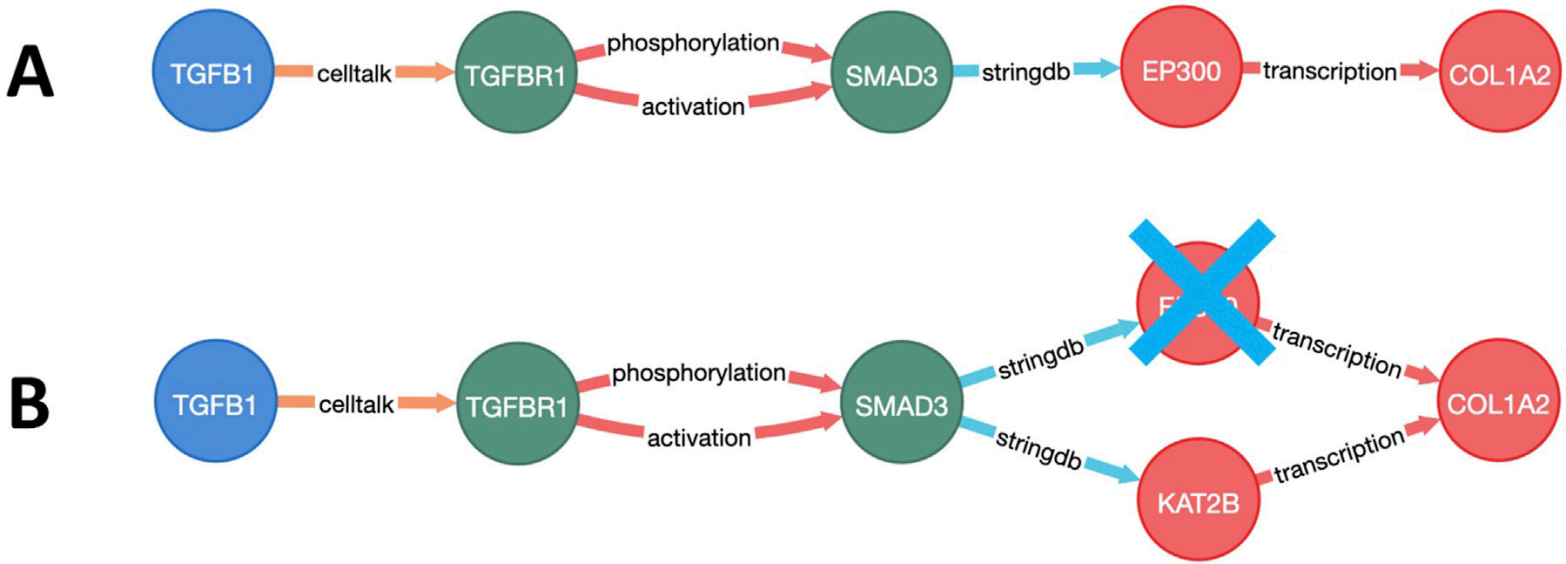
Visualization of a growth factor to the extracellular matrix (GF–ECM) pathway. **(A)** Pathway from GF (TFGB1) to ECM protein (COL1A2). **(B)** Alternative route of the GF–ECM pathway when EP300 is knocked out.

To select the most relevant targets, a multistep approach was employed. First, the weighted and directional fibrosis disease network was constructed (Supplementary Material S1.2). Then, the relative costs of the paths from each of the starting points (GF) to each of the endpoints (ECM) were calculated using Yen’s K-shortest-path algorithm (Figure 2A). Next, each node of the network was individually excluded (*in silico* knockout), and the cost of each pathway from the start to end points with knockout was recalculated (Figure 2B). The top-100 paths that showed the greatest cost increases for each GF–ECM combination were used to identify the top target nodes.

**FIGURE 3:**
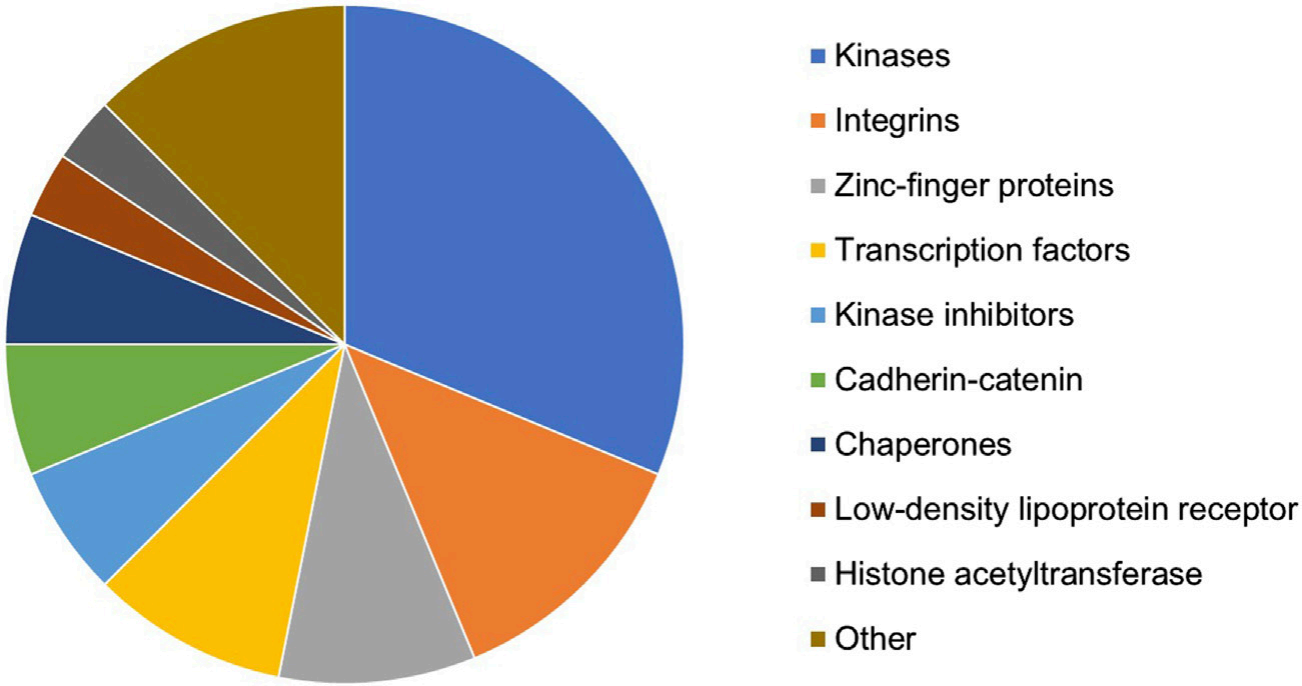
Distribution of target types/families of the 32 proteins resulting from the *in silico* knockout experiment and selected for quick-scan analysis.

In total, 44 targets affecting multiple GF–ECM pathways were selected as the top target candidates for further analyses. Some of these targets (such as EGFR, TGFBR1/2, SMAD3, and TP53) were too generic and hence excluded from the candidate list. The remaining shortlist of 32 proteins belonged to various target types/families (Figure 3). Kinases, integrins, zinc-finger proteins, and transcription factors represented the most populated target types within the selection. All 32 potential targets were subjected to a quick-scan analysis, followed by an in-depth target efficacy assessment for the top-5 ranked targets. The approach followed is illustrated below for one of the targets: the HAT E1A-interacting p300 (EP300; alternatively called KAT3B). The other four targets are currently being explored experimentally for their antifibrotic potential in liver fibrosis.

### 3.3 Target evaluation and selection for EP300

#### 3.3.1 Quick-scan analysis

To distinguish potentially promising candidate targets from those with lesser therapeutic merits, a quick-scan analysis was performed. With the aim of obtaining a quick, global view of a target, database information was queried with a focus on fibrosis and MASH (Supplementary Material S1.3) using the TargetTri system. The key findings for EP300 are listed in Table 2. EP300 has 45 neighbors associated with one of the fibrosis terms, of which six are present in the shortlist of 32 candidate drug targets from *in silico* knockout experiments. Although EP300 itself is only linked to obesity within the spectrum of metabolic and liver diseases, its neighbors are associated with additional phenotypes according to the Comparative Toxicogenomics Database (CTD; Supplementary Material S1.3). These include various forms of fibrosis, liver cirrhosis and cholestasis, fatty liver, dyslipidemias, and liver injury (Table 2). Further support for its potential role in fibrosis was provided by a knockout mouse model reported in the Mouse Genome Informatics (MGI) database (Baldarelli et al., 2024) (Supplementary Material S1.3), which displays decreased fibroblast proliferation, and excerpts from scientific literature (Table 2).

**TABLE 2:** Highlighted results of the EP300 quick scan. In the quick scan, databases (Supplementary Material S1.3) were queried for evidence that support the role of the candidate drug target (EP300) in liver fibrosis. Top-ranked candidate targets were subjected to an in-depth target efficacy assessment by domain experts (Table 3).

Key observations of the quick scan	
Function	• Functions as histone acetyltransferase and regulates transcription via chromatin remodeling (PubMed:23415232, PubMed:23934153, and PubMed:8945521) • Functions as a transcriptional coactivator for SMAD4 in the TGFβ signaling pathway (PubMed:25514493)
Literature	• p300 knockdown disrupted TGFβ- or UPR-induced HSC activation, and pharmacological inhibition of the C/EBPβ-p300 complex decreased TGFβ-induced HSC activation • Consistently, p300 inactivation suppressed TGFβ1-mediated HSC activation and transcription of genes encoding tumor-promoting factors, such as CTGF, TNC, POSTN, PDGFC, and FGF2, as revealed by microarray analyses • Interestingly, although both TGFβ1- and stiffness-mediated HSC activation required p300, comparison of gene expression datasets revealed that transcriptional targets of TGFβ1 were distinct from those of stiffness-p300 mechanosignaling • Fibroblast expression of the coactivator p300 governs the intensity of profibrotic responses to TGFβ • Stimulation of p300 by TGFβ was independent of Smads and involved the early–immediate transcription factor EGR-1 (early growth response 1), which is a key regulator of profibrotic TGFβ signaling • Remarkably, EP300 inhibition reduces fibrotic hallmarks of *in vitro* (patient-derived primary fibroblast), *in vivo* (bleomycin mouse model), and *ex vivo* (precision-cut lung slices, PCLS) IPF models • TGFβ reporter assays demonstrated that p300 mRNA knockdown via targeted siRNAs led to reduced responses to TGFβ, whereas knockdown of CBP by the same approach had an insignificant effect • Overexpression of the transcriptional coactivator p300 rescued TGFβ stimulation of COL1A2 promoter activity in fibroblasts overexpressing p53 • Inhibition of p300 and its binding partners may serve as novel therapies in the treatment of liver diseases
Fibrosis neighbors	45; including 6 in shortlist
Disease pathways^a^	Obesity, fibrosis, IPF, fatty liver, dyslipidemias, CDILI, cholestasis (including experimental, biliary, and extrahepatic)
Murine genetics	Decreased fibroblast proliferation (knockout; MGI:1858020)
Expression profile	Ubiquitous RNA and protein expressions
Tool compound	Inobrodib (inhibitor; phase I/II)

Abbreviations: IPF, idiopathic pulmonary fibrosis; CDILI, chemical and drug induced liver injury.

^a^ Includes disease pathways of the interacting proteins.

To select the candidate targets for further progression into the pipeline, various criteria were used, including the therapeutic rationale, novelty, selectivity/safety, and druggability/assayability. Another criterion was to establish a balanced portfolio of targets, ranging from very exploratory to more investigated targets. At first glance, EP300 appears to be an intermediate candidate target in this spectrum, for which queries based on the initial database and text-mining results have provided sufficient background evidence on its role in fibrosis. Furthermore, it has a large number of fibrotic neighbors, low molecular weight tool compounds (druggability/assayability), and lacks advanced therapeutics in development targeting fibrosis (novelty). Potentially speaking against its selection were the ubiquitous expression (safety), lack of human genetic disease associations with fibrosis, and potentially pleiotropic effects (safety) expected from a HAT as well as associated signaling modes. However, in the quick scan of all 32 proteins, EP300 scored favorably overall and was allowed to the next stage.

#### 3.3.2 Target efficacy assessment of EP300

**FIGURE 4:**
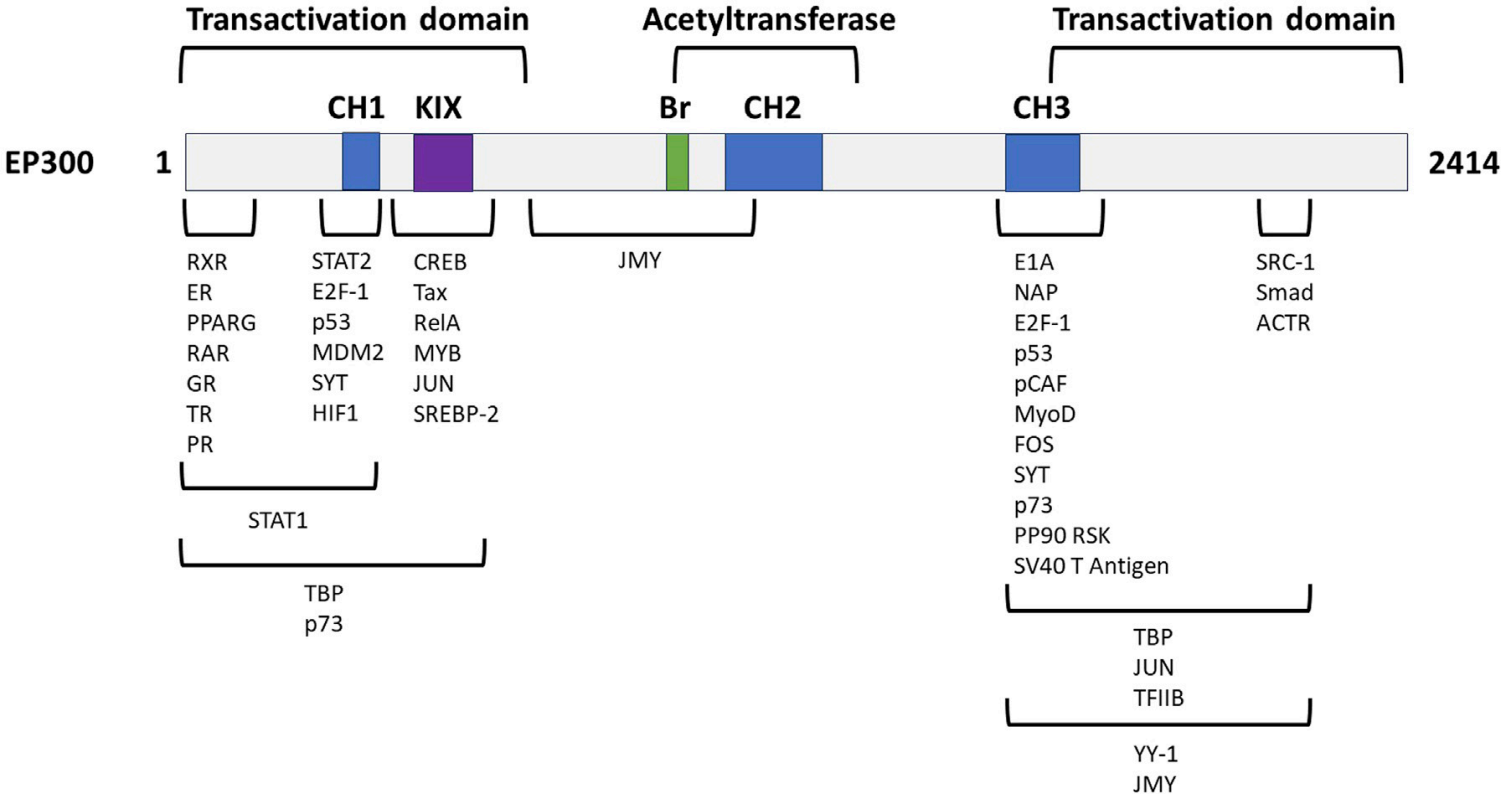
Genetic structure of EP300 with its functional domains and exemplified interaction partners. CH, cysteine-/histidine-rich domain; KIX, kinase inhibitory domain, Br, bromodomain. Adapted from Chan and La Thangue (2001).

To further assess the therapeutic potential of EP300 in MASH-related liver fibrosis, the criteria listed in Table 3 were analyzed in greater depth. The full analysis is shown in Supplementary Material S2. The general characteristics of the target were investigated first, and literature evaluations showed that EP300 is an evolutionary conserved gene belonging to the EP300/CBP (CREB-binding protein or KAT3A) family of proteins, which function as transcriptional coactivators and are involved in multiple, signal-dependent transcription events (Chan and La Thangue, 2001). Both proteins are present in humans as well as most higher eukaryotes and represent HAT enzymes that specifically acetylate H3K27. Because of their high sequence homology, CBP and EP300 are collectively referred to as p300/CBP (Yao et al., 2018). Despite this fact, EP300 and CBP are not fully functionally redundant as differences have been reported with respect to their protein partners, substrate specificity, and selectivity based on enzyme levels, and cell-type specific functions, with both proteins considered as requisites for mammalian development (Chan and La Thangue, 2001; Vo and Goodman, 2001; Martire et al., 2020; Turnell, 2015). The N- and C-terminal domains of EP300 act as transactivation domains, interacting with a wide range of proteins to form various transcriptional complexes; the acetyltransferase domain is located in the central region of the protein (Figure 4) (Chan and La Thangue, 2001; Ghosh and Varga, 2007). Physiologically, EP300 is involved in cellular processes such as proliferation, migration, differentiation, senescence, and apoptosis through chromatin remodeling in the regulatory regions of the genes. In these cellular processes, EP300 acts as an epigenetic regulator and/or an interacting coactivator with specific transcription factors of genes (Ghosh, 2021).

**TABLE 3:** Evidence in support of the roles of EP300 in MASH-related fibrosis obtained by expert-based in-depth target efficacy assessment. The key findings derived from literature and other data sources are shown. The full EP300 efficacy assessment is available in Supplementary Material S2.

Criterion	Evidence	References
Target characteristics	• Histone acetyltransferase • Transcriptional coactivator • Related to but not redundant with CBP	Ghosh (2021); Chan and La Thangue (2001)
Therapeutic rationale for fibrosis	Wnt/β-catenin signaling has been associated with organ fibrosis. Nuclear β-catenin recruits CBP or p300 to stimulate the transcription of its target genes TGFβ stimulates COL1A2 transcription via functional cooperation between Smad3 and p300/CBP transcriptional coactivators The HIF-1α-p300/CBP complex binds to the hypoxia response element (HRE) to activate the transcription of genes involved in fibrogenesis	Yao et al. (2018); Inagaki et al. (2003)
Therapeutic rationale for MASH	EP300 mediates SREBP-1c acetylation. Elevated SREBP-1c acetylation has been associated with increased lipogenic gene expression EP300 acetylates FXR, resulting in activation of its target genes. FXR acetylation is constitutively elevated in metabolic disease states EP300 acetylates ChREBP, a transcriptional activator of glycolytic and lipogenic genes EP300 acetylates HNF4, which is involved in lipid homeostasis and glucose metabolism among others Transcriptional activation of NF-кB, which induces inflammatory responses, requires association with CBP/p300	Ponugoti et al. (2010); Kemper et al. (2009); Bricambert et al. (2010); Tsai et al. (2017); Zhong et al. (2002)
Expression profile	Expression of p300 was increased in the livers of mice following CCl_4_ injection The p300-C/EBPα/β pathway is activated in the livers of patients with NAFLD. Elevated levels of p300 or its mutant forms are associated with skin, lung, and cardiac fibrosis	Dou et al. (2018); Jin et al. (2013); Ghosh (2021)
Human genetic phenotype	Not available	—
Animal genetic phenotype	HSC activation and collagen deposition were reduced in the livers of p300^F/Fcre^ (i.e., knockout) mice given intraperitoneal injections of carbon tetrachloride compared with control mice Livers of transgenic mice expressing a dominant negative p300 molecule (dnp300) were resistant to CCl_4_-mediated injury and showed reduced apoptosis but increased proliferation after injury Portal pressure and liver fibrosis were reduced in mice with liver sinusoidal-endothelial-cell-specific p300 deletion (p300^LSECΔ/Δ^ mice) compared to p300 ^fl/fl^ control mice following liver injury Transgenic mice expressing dominant-negative p300 had fewer C/EBPα/β-p300 complexes and did not develop age-dependent hepatic steatosis	Dou et al. (2018); Breaux et al. (2015); Gao et al. (2021); Jin et al. (2013)
Competitive landscape	Several small-molecule inhibitors in (pre)clinical development, mainly to treat cancer Inobrodib (CCS-1477) is in the highest phase of development (phase I/II)	Cortellis, Supplementary Material S2
Medicinal chemistry tools	Commercially available inhibitors, siRNA, and shRNA Functional readout described: gene expression; HSC activation Chemiluminescent and binding assays (FRET) for p300 inhibition are available/have been described	Supplementary Material S2
Preclinical evidence	Multiple *in vitro* (human LX-2; normal and activated rat HSCs) and *in vivo* (transgenic and CCl_4_-treated mice) studies supporting the roles of EP300 in liver fibrosis are reported in literature Studies supporting the roles of EP300 in non-liver fibrosis, lipid metabolism/NAFLD, and inflammation	Supplementary Material S2
Clinical evidence	Not available	—

One of the important criteria in evaluating a candidate drug target is its therapeutic rationale. Here, the mechanistic role of the target in disease pathogenesis, i.e., MASH-related liver fibrosis, is considered. As MASH is a multifactorial disease with triggers such as dyslipidemia, insulin resistance, inflammation, and oxidative stress, the roles of EP300 in these aspects of the disease were also explored. As shown in Table 3, EP300 is implicated in fibrogenesis through various mechanisms; it is also associated with processes that are dysregulated in MASH. The roles of EP300 in liver fibrosis were further supported by *in vitro* and *in vivo* studies (Supplementary Material S2). For example, liver fibrosis was reduced in CCl_4_-treated EP300 knockout mice (Gao et al., 2021; Breaux et al., 2015; Dou et al., 2018).

#### 3.3.3 *In vitro* validation of EP300

**FIGURE 5:**
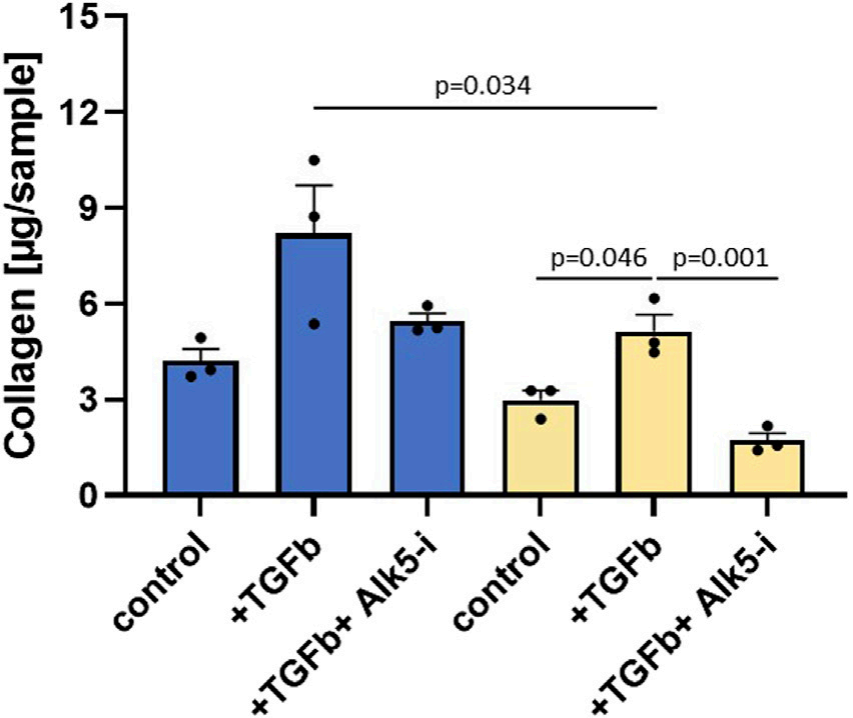
Silencing RNA (siRNA) targeting EP300 shows a significant (*p*-value < 0.05) reduction in fibrosis. Total collagen levels measured in primary hepatic stellate cells (HSCs) in the presence of siRNA targeting either eGFP (blue, control siRNA) or EP300 (yellow). Aside from the untreated controls, the cells were stimulated with TGFβ (+TGFβ) in the absence or presence of the ALK5 inhibitor (+TGFβ+Alk5-i). The data are presented as mean ± SD (n = 3).

To validate the regulatory role of EP300 in liver fibrosis, siRNA and compound intervention studies were performed. TGFβ that transduces its signal through the ser/thr kinase receptor ALK5 was used to induce fibrosis. ALK5 inhibition served as the reference for maximal inhibition of TGFβ signaling resulting from both external stimuli and cellular autoactivation. TGFβ-induced transcription of genes, including collagens and PAI-1, affects protein synthesis and consequently the total protein levels. Therefore, ALK5 inhibition can also be used to determine the effects of TGFβ on protein synthesis. The total protein levels above and below those observed with ALK5 inhibition are respectively indicative of protein synthesis inhibition and cellular toxicity. As illustrated in Figure 5, siRNA targeting EP300 in human primary stellate cells was able to significantly reduce the total collagen level after TGFβ stimulation as compared to the eGFP siRNA control (*p* = 0.034). Addition of the ALK5 inhibitor (Alk5-i) further reduced total collagen (*p* = 0.001), showing that siRNA was not able to inhibit TGFβ signaling completely.

**FIGURE 6:**
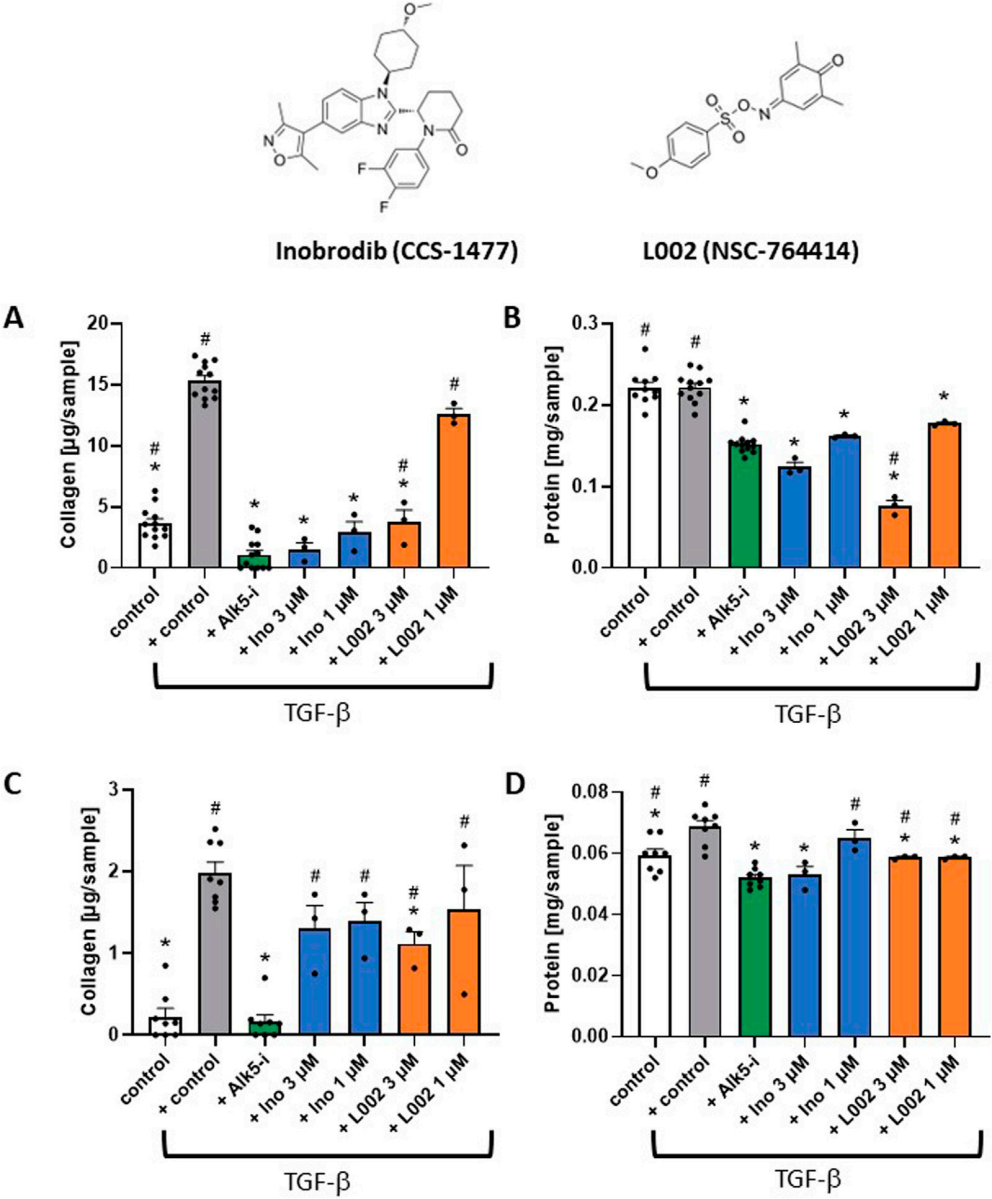
Intervention studies with small-molecule inhibitors of EP300 (inobrodib and L002) as a case study for target validation. **(A)** Inobrodib (Ino) and L002 significantly decrease TGFβ-induced fibrosis in LX-2 cells. The total collagen levels for treatment with Ino, L002, and ALK5 inhibitor (Alk5-i) are shown after TGFβ stimulation compared to the non-stimulated control. **(B)** Corresponding total protein levels. **(C)** Ino, L002, and Alk5-i, decreased TGFβ-induced fibrosis in primary HSCs. The total collagen levels for treatment with Ino, L002, and Alk5-i are shown after TGFβ stimulation compared to the non-stimulated control. **(D)** Corresponding total protein levels. The data are presented as mean ± SD (n ≥ 3). Significance (*p* < 0.05) is indicated with respect to the TGFβ-stimulated control (*) or Alk5-i (#).

Intervention studies for EP300 were performed with two small-molecule inhibitors, namely, inobrodib and L002 (Figure 6), in both primary HSCs and LX-2 cells. Inobrodib is a potent and selective bromodomain EP300/CBP inhibitor that is currently in clinical development for hematological malignancies (Nicosia et al., 2023). L002 is a nonselective inhibitor of EP300 with an IC50 of 1.98 μM, which additionally binds lysine acetyltransferase 2A and 2B (KAT2A and KAT2B, respectively) (Yang et al., 2013). In LX-2 cells, the inhibitors significantly reduced the total collagen levels at concentrations of 1 μM (inobrodib) and 3 μM (inobrodib and L002), with inobrodib showing collagen inhibition levels similar to those of Alk5-i (Figure 6A). The total protein levels were also reduced with respect to both the stimulated and non-stimulated control, indicating inhibition of protein synthesis due to reduced transcription. At an L002 concentration of 3 μM, the protein level was lower than that observed with Alk5-i, indicating cellular toxicity (Figure 6B). In primary HSCs, a significant reduction of total collagen level was observed for L002 at a concentration of 3 μM (Figure 6C). Administration of 1 μM of L002 as well as 1 μM and 3 μM of inobrodib resulted in lower total collagen levels. As observed in the LX-2 cells, at higher concentrations (>10 µM), both inhibitors showed more pronounced effects on lowering the total collagen with significant cellular toxicities (data not shown). At inhibitor concentrations of 1 and 3 μM, the total protein levels were higher than or similar to those observed with Alk5-i (Figure 6D). Therefore, neither inhibitor showed toxic effects at these concentrations. Altogether, the siRNA results and results obtained with two small-molecule inhibitors tested on HSCs and LX-2 cells confirm the causal role of EP300 in liver fibrosis, which is in agreement with the results from our weighted, directional data-driven disease network. Accordingly, the *in vitro* experiments validate our novel network approach.

## 4 Discussion

The present study clearly shows the utility of our mechanism-based, data-driven approach in identifying novel candidate drug targets for complex diseases, such as MASLD. In our experimental validation of *in silico* target selection, we confirmed EP300 as a gene relevant to liver fibrosis. For siRNA targeting EP300, a significant reduction of 37% of the total collagen was observed. Next, intervention studies using two small-molecule inhibitors targeting EP300 confirmed the results obtained from the siRNA studies. Inobrodib and L002 were able to reduce the total collagen levels to varying extents in both LX-2 cells and primary HSCs (Figure 6). The efficacies of these compounds have not been demonstrated previously in functional *in vitro* liver fibrosis assays. The experimental results thus substantiate the governing role of EP300 in liver fibrosis. A recent report on EP300, which was published toward the end of our study, further supports this observation (Rubio et al., 2023), as do the *in vivo* observations uncovered during our target efficacy analysis, showing that EP300 knockout or knockdown can reduce liver fibrosis and its underlying processes (Supplementary Material S2). Hence, this proof-of-concept exploration successfully demonstrates the ability of our data-driven approach to identify novel candidate drug targets that play crucial roles in the molecular mechanisms of diseases.

Our generic data-driven pipeline for mechanism-based target discovery allows the construction of a directional disease-specific network, its enrichment and weighing with (pre)clinical data, and its mechanistic exploration with *in silico* knockouts to identify candidate drug targets. This approach is unique in that it integrates multiple features of clinical relevance that are crucial for generating a disease network. For instance, the network directionality ensures adherence to physiologically relevant signaling cascades. This is strengthened by the inclusion of clinical MASH data that not only boost the translational power but also ensure identification of the most relevant pathways for liver fibrosis. Finally, scrutiny of these pathways at a mechanistic level via *in silico* knockouts ensures that much-needed insights into the molecular aspects of MASH are obtained and that human-disease-mechanism-relevant drug targets can be identified.

By leveraging the power of text mining, transcriptomics, and biological networks in our approach, the major challenges in drug discovery are tackled. One of these challenges is the higher complexity and lower chance of success of drug development given the recent focus on complex diseases (Sun et al., 2022; March et al., 2021). Another challenge is the relentless growth of data, notably textual information, and its unstructured nature. Unstructured data represent valuable sources that are frequently untapped, resulting in unnecessary data gaps. The implementation of artificial-intelligence-based technologies, as in this work, can assist in overcoming these hurdles. Natural language processing (NLP) strategies have enabled the shift from time-consuming manual data curation and interpretation to large-scale automated text analyses (Jin et al., 2024). Biological network and transcriptomics strategies have also instigated a move from phenotypic disease management to mechanism-based disease curation (Nogales et al., 2022; Barabási et al., 2011).

The merits of disease or pharmacological networks in drug development have been demonstrated in several studies. However, these studies generally focus on identifying targets for active compounds (Sakle et al., 2020) or heavily rely on disease-associated genes available in databases (Quan et al., 2021). Our approach differs from these studies in the construction of a disease network as it does not rely on curated annotations in databases at the onset (e.g., gene cards and OMIM) or on human disease transcriptomics datasets available in the public domain. Instead, it applies disease-agnostic text-mining strategies using customized ontologies, whose benefits include the ability to perform queries using terms that are not part of the controlled vocabularies used in databases and to zoom-in on specific aspects or features of the contributing disease processes. For example, inflammation is a contributing factor of fibrosis. However, inflammation is a rather general process when considering therapeutic interventions. In such a case, only information pertaining to liver-specific inflammation can be included. Alternatively, customized ontologies can be applied to exclude certain types of data or disease subtypes that are of lesser interest, e.g., kidney fibrosis, as used in the current study. Text mining also allows the inclusion of emerging data; with our PubMed processing pipeline that performs daily updates (Venhorst and Kalkman, 2024), off-the-press observations are also taken into account.

Despite the success of our approach, several limitations need to be considered. For less explored diseases and proteins, a general pitfall for any approach involving biological networks is data availability that limits the network capabilities. However, new methods are continuously becoming available to mitigate these shortcomings and allow access to novel data sources. For example, advancements in sequencing archived formalin-fixed paraffin-embedded (FFPE) materials, as demonstrated by us recently (Verschuren et al., 2024), could enhance the approaches described in the present study. In terms of target evaluation, highly exploratory candidate drug targets often lack public information that impacts the results of the quick-scan and in-depth target evaluations. For such high-risk targets, it is crucial to have a well-defined therapeutic rationale. Another potential limitation pertains to text mining. We use text mining as the first step in selection in the proposed approach, so some basic knowledge on the hallmarks or mechanisms of disease is necessary to create a fit-for-purpose taxonomy. The use of such a custom taxonomy may result in user bias when selecting the terms to be used, impacting the outcomes of text mining. Therefore, the taxonomy needs to be broad enough to capture all relevant aspects of the disease, ranging from molecular processes to clinical observations, to identify not only emerging and novel proteins but also known genes. The recall of an early MASH-related fibrotic signature was included in this work to assess the robustness of our text-mining strategy. Alternatively, transcriptomics or genome-wide association study (GWAS) data derived from other sources (see below) can be combined with text mining to construct an initial disease network. In this study, we used clinical transcriptomics data as the external source to validate and weight the liver fibrosis disease network as well as increase its translational value.

To explore the network and identify hub genes governing disease progression, we defined starting nodes based on molecular initiating events of the disease (i.e., GFs) and distinct but clinically relevant end points as the end nodes (i.e., collagens), which can also be used as readouts during *in vitro* target validation. The translational power was boosted by adding clinical data to weight the edges. It should be noted that the proposed approach is not a requirement for target identification. For example, seed nodes close to a pathological gene can also be used (Gentili et al., 2022). However, the directed and weighted approach combined with clinically relevant data described herein enables identification of a more causal disease pathway and consequently more relevant candidate drug targets. Moreover, this directed and weighted approach can be applied to target specific patient groups in the future for precision medicine using patient-specific transcriptomics data. For example, the phenotype of MASH varies widely across individuals (Harrison et al., 2023), even at the level of HSCs and their activation (Bogomolova et al., 2024). These phenotypes likely involve divergent pathological pathways (González Hernández et al., 2024). A single drug is unlikely to be effective for the entire population, and this is highlighted by the fact that the first drug (resmetirom) was approved by the US FDA only in March this year and that only 25%–30% of patients benefit from treatment with this drug (Harrison et al., 2024), further stressing the unmet medical need for patients suffering from MASH.

As indicated above, the future directions of the proposed approach include the construction of patient-subgroup-specific disease networks to capture disease heterogeneities. Novel techniques, such as single-cell sequencing, and multi-omics integration are expected to further facilitate this progress (Du et al., 2024). Another challenge in treating liver fibrosis is the slow disease progression, where the disease develops over an extended period of time. The inclusion of temporal processes in the disease network, e.g., using omics data obtained during disease progression at different time points, may provide a clearer view on the processes at play at various stages of the disease and aid in the identification of successful drug targets. Finally, developments in artificial intelligence technologies may further advance drug-discovery efforts. In terms of text mining, for example, more precise definitions of the causality of protein–effect relations using LLMs can further assist in more accurate rankings of the key players in the specific pathological processes under investigation (Venhorst and Kalkman, 2024). As a next step in our target discovery effort, we intend to continue the validation of our candidate drug targets. This notably includes *in vivo* validation of the targets in relevant disease models. In addition, the applicability of our approach will be confirmed in disease indications other than liver fibrosis to demonstrate its generic utility.

## 5 Conclusion

In this work, we present the development of a data-driven approach that integrates text mining with network models for target identification based on mechanistic disease insights. The directional and weighted approach presented herein not only identifies causal disease pathways but also potentially paves the path to discovering patient-specific disease pathways and their corresponding interventions. The proposed pipeline was successfully validated with a proof-of-concept study using EP300 as the exemplar novel candidate drug target for MASH-induced liver fibrosis. To fully leverage the potential of our approach, further validation and subsequent drug-discovery activities are warranted for the identified targets. Aside from functional studies, this includes mechanistic validation of target engagement (binding), hit-to-lead programs, ADMET studies, and eventual *in vivo* studies. As the approach described here is disease agnostic, we believe that it can significantly contribute to successful drug-discovery programs focused on diseases other than MASH-related liver fibrosis.

## Data Availability

The original contributions presented in the study are included in the article/Supplementary Material, and any further inquiries may be directed to the corresponding author.
