# Supplementary material for: Integrating text mining with network models for successful target identification: in vitro validation in MASH-induced liver fibrosis
Source: Front Pharmacol. 2024 Sep 27;15:1442752. doi: 10.3389/fphar.2024.1442752 (PMC11466758; doi:10.3389/fphar.2024.1442752)
Supplement: Supplementary file 1 [file DataSheet2.PDF]

Supplementary material 2

Integrating Text-Mining and Network Models for Successful Target Identification: Validation in MASH-induced liver fibrosis

Jennifer Venhorst[1*], Roeland Hanemaaijer[2], Remon Dulos[3], Joline Attema[2], Karin Toet[2], Christa de Ruiter[2], Gino Kalkman[1], Tanja Rouhani-Rankhoui[1], Lars Verschuren[3]

[1]Biomedical and Digital Health, The Netherlands Organization for Applied Scientific Research (TNO), Princetonlaan 6, Utrecht, The Netherlands.

[2]Department of Metabolic Health Research, The Netherlands Organization for Applied Scientific Research (TNO), 2333 CK Leiden, The Netherlands.

[3]Department of Microbiology and Systems Biology, The Netherlands Organization for Applied Scientific Research (TNO), 2333 CK Leiden, The Netherlands.

*** Correspondence:**
Jennifer Venhorst
Jennifer.mccormack@tno.nl

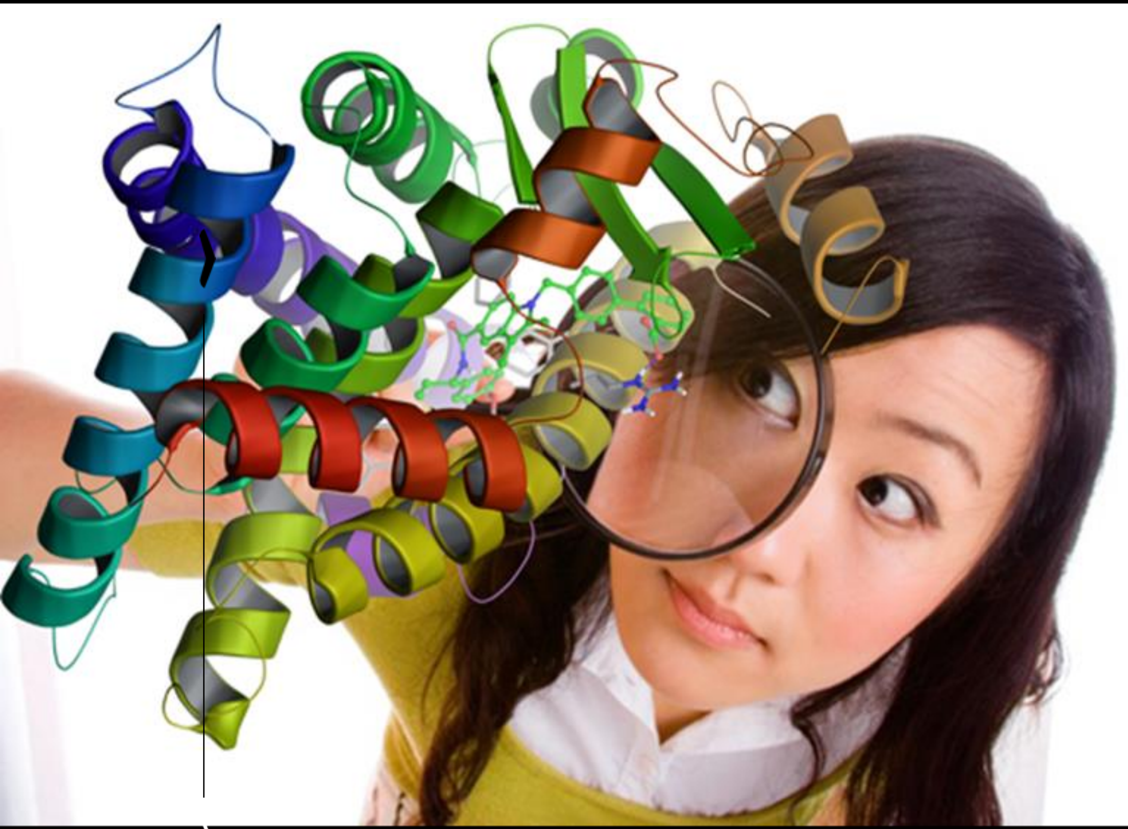

Target Evaluation

Target: EP300

MoA: Inhibition

Indication: Liver fibrosis, NAFLD

Date: 21 February 2022

Jennifer McCormack-Venhorst
Tanja Rouhani Rankouhi

CONTENTS

- **Disease characteristics**
- Target characteristics
- Therapeutic rationale
- Expression profile
- Human genetic phenotype
- Animal genetic phenotype
- Competitive landscape
- Medicinal chemistry tools
- Preclinical evidence
- Clinical evidence

LIVER STAGES IN HEPATIC FIBROSIS

- **Figure. Distinct cellular landscapes characterize homeostasis, regeneration, fibrosis, and resolution in the liver**. The homeostatic liver is characterized by rare cell proliferation and lack of de novo extracellular matrix (ECM) deposition. During regeneration, epithelial replacement occurs predominantly via hepatocyte proliferation and, to a minor degree, through the activation of ductal progenitors. Resident (Kupffer) and bone marrow–recruited macrophages phagocytose the dead epithelium and launch an inflammatory cascade (e.g., TNF-α, IL-6). CXCR7/CXCR4+ LSECs provide mitogenic signals (HGF, WNT2) that sustain hepatocyte proliferation. Hepatic stellate cells (HSCs) transdifferentiate into myofibroblasts that deposit ECM on the wound site, although this matrix can be degraded via metalloproteinases (MMPs). In fibrosis, the hepatocyte compartment is highly senescent and ductal progenitor expansion becomes predominant. Monocyte-derived Ly6Chi macrophages (secreting TGF-β, thrombospondin 1) and CXCR4+ LSECs (secreting TGF-β, BMP2, and PDGFC) collectively enhance myofibroblast proliferation and survival. Myofibroblasts, in turn, secrete high levels of tissue inhibitors of metalloproteinases (TIMPs), which inhibit metalloproteinases (MMPs) and cause excessive matrix accumulation. A Th1- versus Th2-skewed immune system favors regeneration versus fibrosis, respectively. The resolution of fibrosis entails the return to quiescence/inactivation of myofibroblasts as well as their clearance by NK cells, γδ T cells, and Ly6Clo macrophages. High levels of MMPs contribute to matrix degradation. The mechanisms of epithelial replacement at this stage have not been fully elucidated (Cordero-Espinoza, 2018).

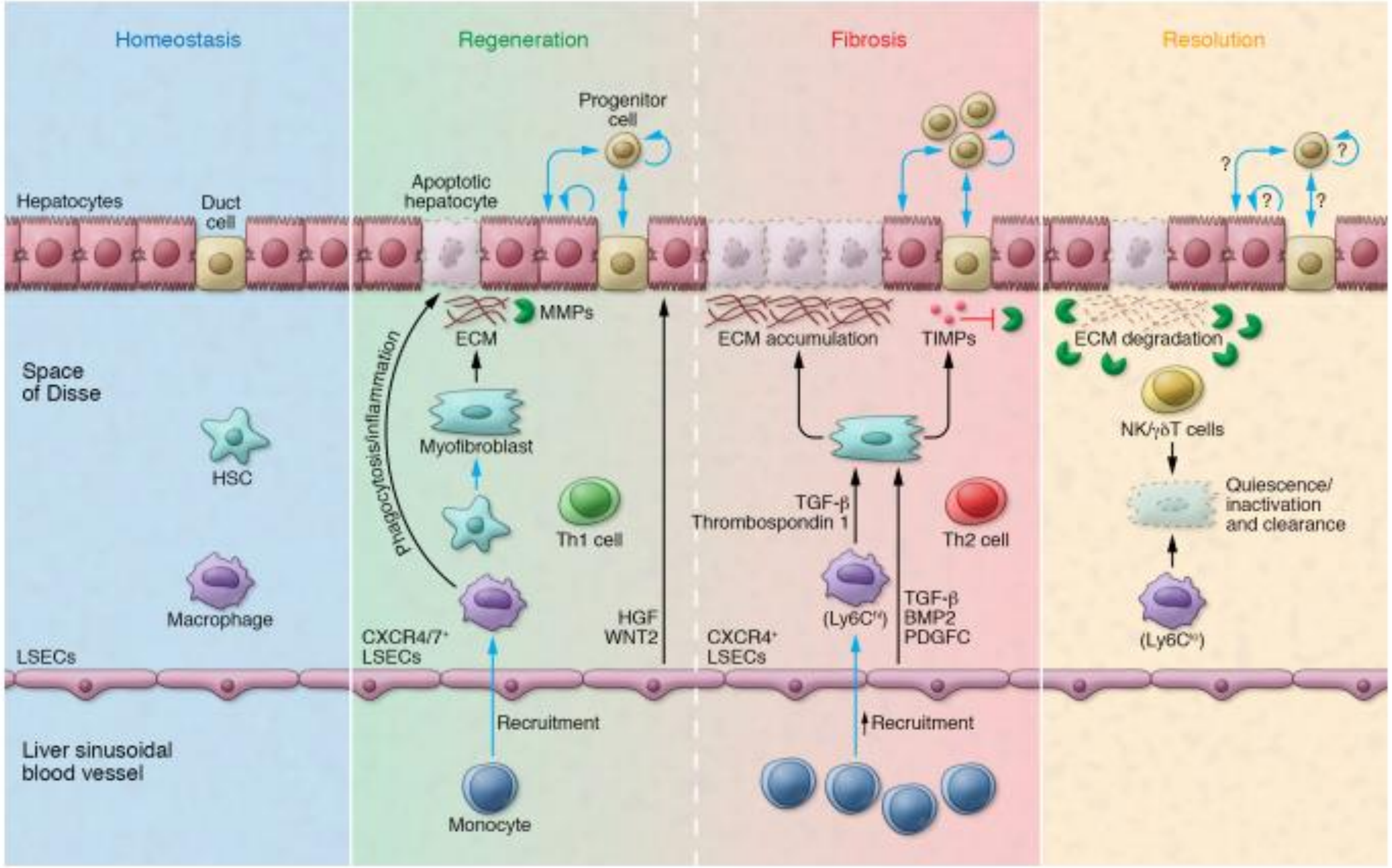

HEPATIC FIBROSIS

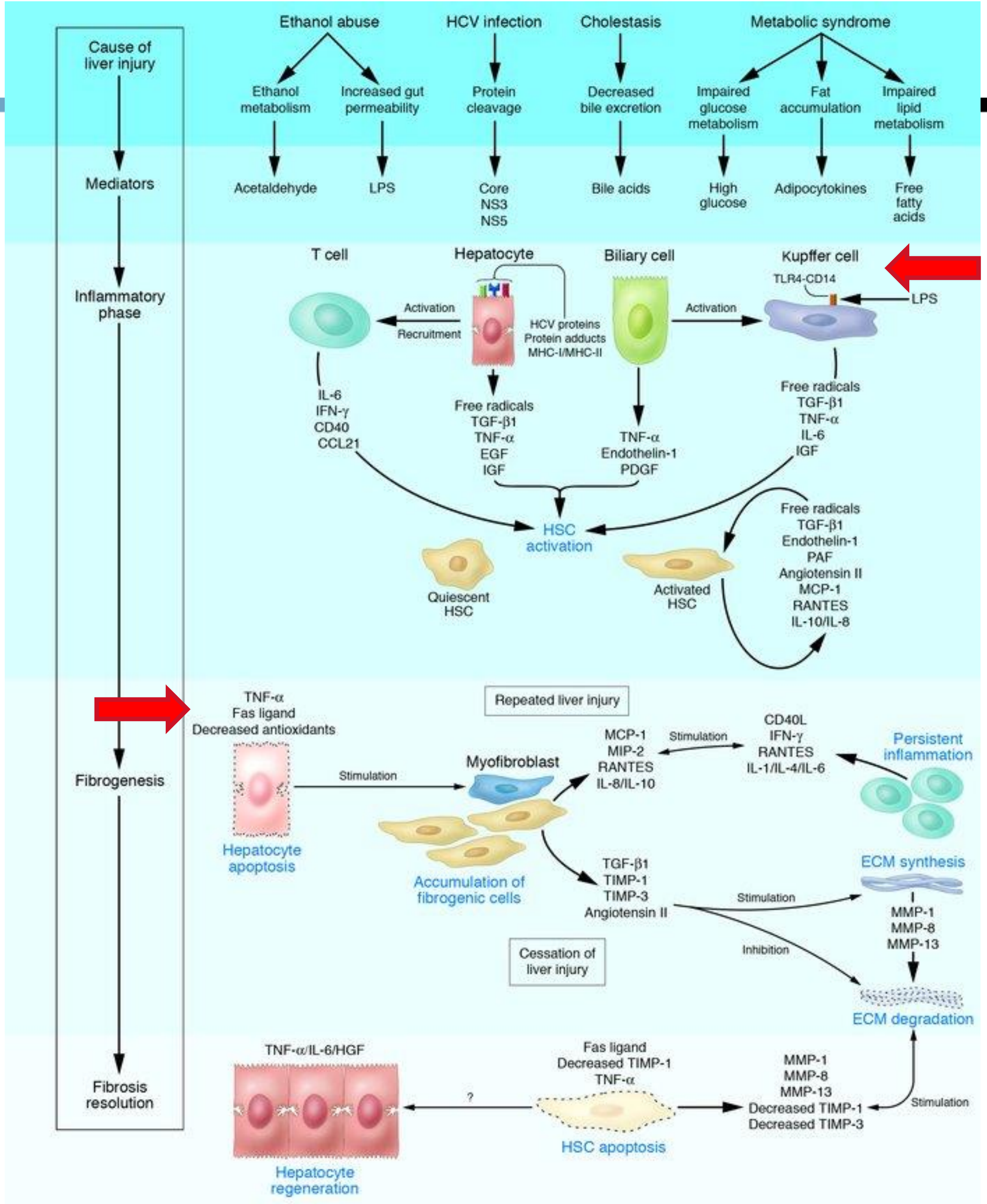

Figure: Cellular mechanisms of liver fibrosis. Different types of hepatotoxic agents produce mediators that induce **inflammatory actions** in hepatic cell types. Damaged hepatocytes and biliary cells release inflammatory cytokines and soluble factors that activate Kupffer cells and stimulate the recruitment of activated T cells. This **inflammatory milieu stimulates** the activation of resident hepatic stellate cells (HSCs) into **fibrogenic myofibroblasts**. Activated HSCs also secrete cytokines that perpetuate their activated state. If the liver injury persists, accumulation of activated HSCs and portal myofibroblasts occurs, synthesizing large amounts of extracellular matrix (ECM) proteins and leading to tissue fibrosis. ECM degradation is inhibited by the actions of cytokines such as tissue inhibitors of metalloproteinases (TIMPs). **Apoptosis** of damaged **hepatocytes stimulates the fibrogenic actions** of HSCs. If the cause of the liver injury is removed, fibrosis is resolved. This phase includes apoptosis of activated HSCs and regeneration of hepatocytes. Collagen is degraded by increased activity of metalloproteinases (MMPs) induced by decreased TIMP expression. CCL21, C-C chemokine ligand 21; MCP-1, monocyte chemoattractant protein–1; MIP-2, macrophage inflammatory protein–2; NS3, HCV nonstructural protein 3; NS5, HCV nonstructural protein 5; PAF, platelet-activating factor (Bataller, 2005).

CONTENTS

- Disease characteristics
- **Target characteristics**
- Therapeutic rationale
- Expression profile
- Human genetic phenotype
- Animal genetic phenotype
- Competitive landscape
- Medicinal chemistry tools
- Preclinical evidence
- Clinical evidence

TARGET CHARACTERISTICS

- E1A-interacting p300 gene (Ep300 or p300) is an evolutionarily conserved gene and has been cloned and characterized in different species (Ghosh, 2020). It belongs to the p300/CBP proteins, which include the distinct but related proteins p300 and CBP and other proteins, such as p270. Viral oncoproteins, such as adenoviral E1A and SV40 large T antigen, specifically target these proteins (Chan, 2001).

- p300 and CBP are two acetyltransferase enzymes in humans and most higher eukaryotes. p300 (also called EP300 or KAT3B) is so-named because it is about 300 kDa in size (with 2414 amino acids). CBP (also called CREBBP or KAT3A) is composed of 2441 amino acids, and because of the high sequence homology observed between it and p300, the two proteins are collectively referred to as p300/CBP (Yao, 2018).

- Histone acetyltransferases acetylate histone tails and numerous transcription factors and thus controls target gene expression through chromatin organization as well as activity of the transcription factors (Ghosh, 2020).

- P300 has several functional domains—three cysteine/histidine rich (C/H) domains, an acetyltransferase (AT) domain, a bromodomain, and a glutamine rich (Q) C-terminal domain—that interact with numerous transcription factors in a context-dependent manner (Ghosh, 2020).

- Since the cloning of Ep300 gene and its molecular characterization, numerous studies have documented the significant physiological role of p300 in maintenance of cellular homeostasis and organismal health through specific interaction with transcription factors, cofactors and signaling molecules (Ghosh, 2020).

TARGET CHARACTERISTICS

- Acetyltransferase p300 plays significant roles in numerous cellular processes including proliferation, migration, differentiation, senescence, and apoptosis through chromatin remodeling in the regulatory regions of genes as an epigenetic regulator and/or an interacting coactivator with specific transcription factors of genes involved in those cellular processes (Ghosh, 2021).

- Significantly **elevated levels of p300 or its mutant forms are associated with skin, lung, and cardiac fibrosis** (Ghosh, 2020).

- Under normal growth conditions, fibroblasts synthesize and secrete low levels of extracellular matrix protein type I collagen using basal transcriptional machinery. However, when fibroblasts are exposed to pleiotrophic cytokine transforming growth factor-beta (TGF-β), activated TGF-β receptor complex kinase phosphorylates cytoplasmic R-Smad2/3 that is followed by the interaction of pSmad2/3 with Co-Smad4 and translocation of Smad2/3-Smad4 heterodimers into the nucleus. **Activated Smad2/3/4 complex binds to Smad-binding element of collagen promoter and interacts with and recruits acetyltransferase p300 to the transcriptional complex**. Activated transcriptional complex significantly **increases the transcription of collagen gene** (Ghosh, 2021).
  - Presence of acetyltransferase p300 or unphosphorylated Smad proteins in unstimulated cells are not sufficient to activate target gene expression, because signal specific modifications of p300 and its interacting factors are required for protein-protein interaction, nuclear translocation, and stimulation of target gene transcription.

- The **target gene specificity of** acetyltransferase **p300** is **controlled** by signal **specific posttranslational modifications** viz. phosphorylations by different kinases, autoacetylation, and methylation by coactivator-associated arginine methyltransferase 1 in a cell type and context dependent manner (Ghosh, 2021).

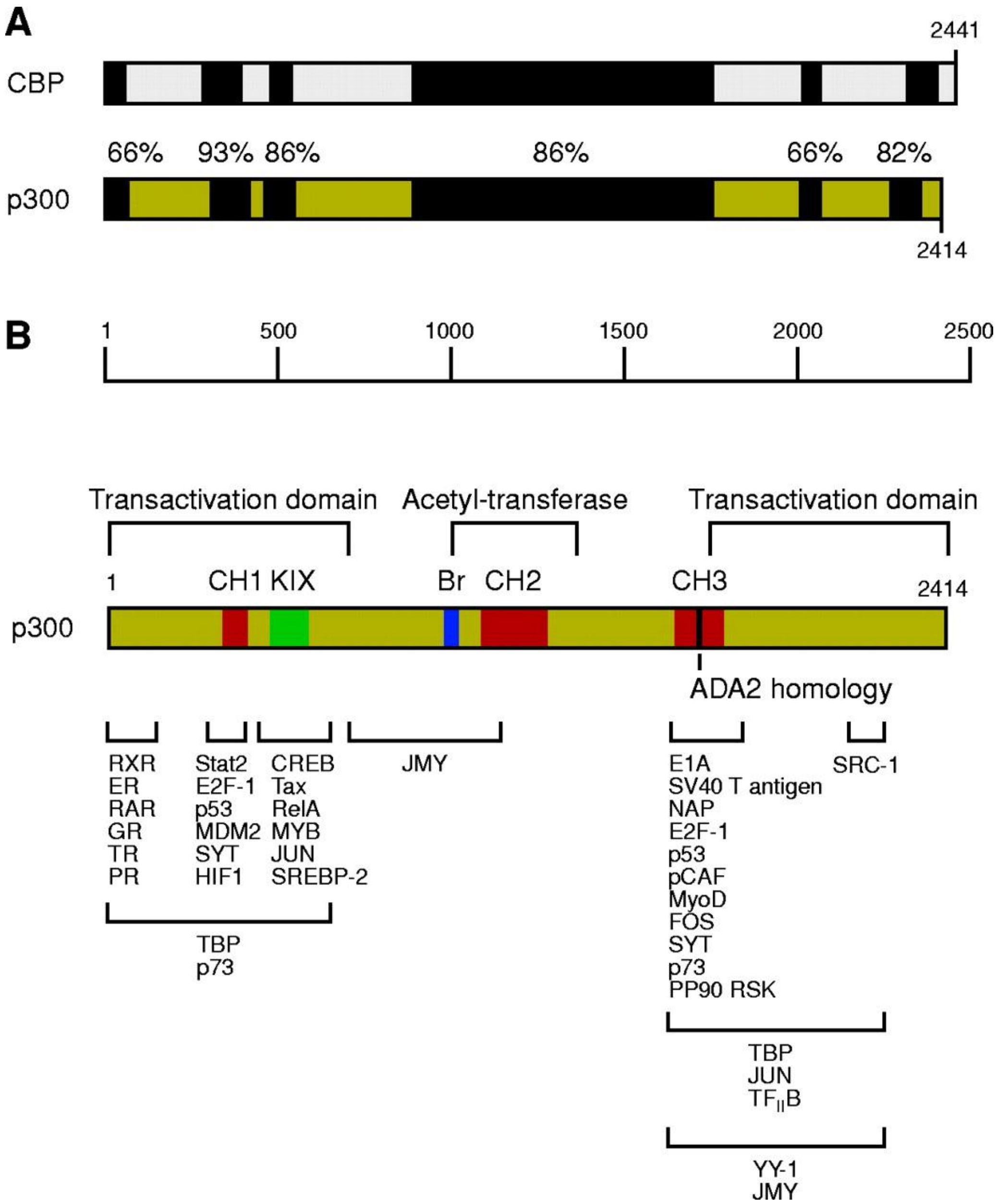

Figure: Organisation of p300/CBP proteins. (A) Comparison of p300 and CBP. The dark regions indicate the areas of highest homology, with the percentage amino acid identity between the two proteins indicated. The size of each protein, in number of amino acid residues, is indicated. (B) The functional domains in p300 are indicated, including the cysteine/histidine-rich domains CH1, CH2 and CH3, the KIX domain, the bromodomain (Br) and the ADA2 homology region. The N- and C-terminal domains of p300/CBP can act as transactivation domains, and the acetyl-transferase domain is located in the central region of the protein. The regions that have been shown to bind to target proteins, together with the identity of the interacting proteins, are shown (Chan, 2001).

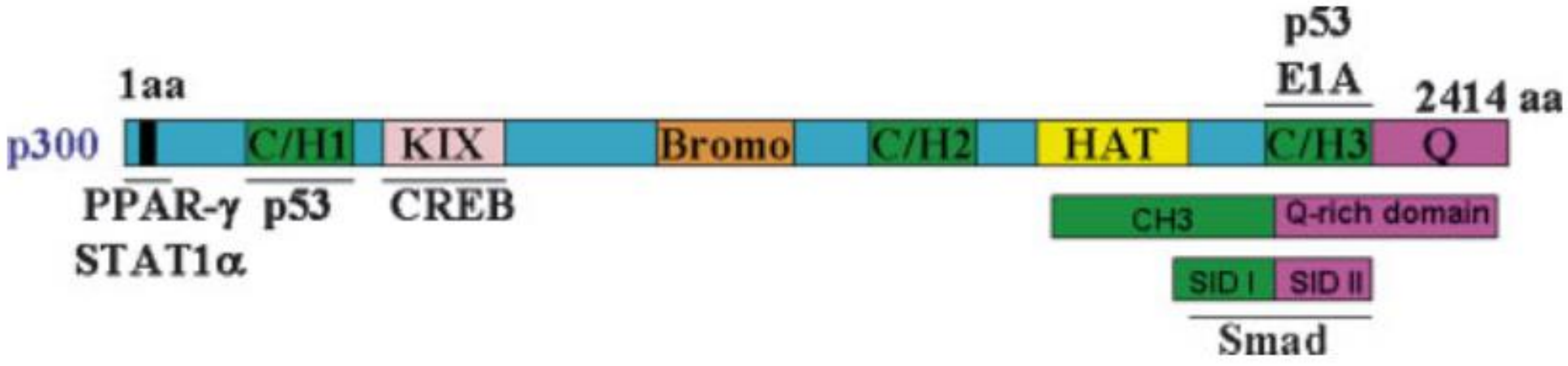

Figure: Functional domains of p300. CH: cysteine/histidine-rich; KIX, kinase inhibitory domain; Bromo, bromodomain; HAT, histoneacetyltransferase; Q, glutamine-rich. SID, Smad interacting domain. aa, amino acid. Interaction sites for peroxisome proliferator-activated receptor-gamma, p53, STAT1a, CREB, E1A, and Smad are shown (Ghosh, 2007).

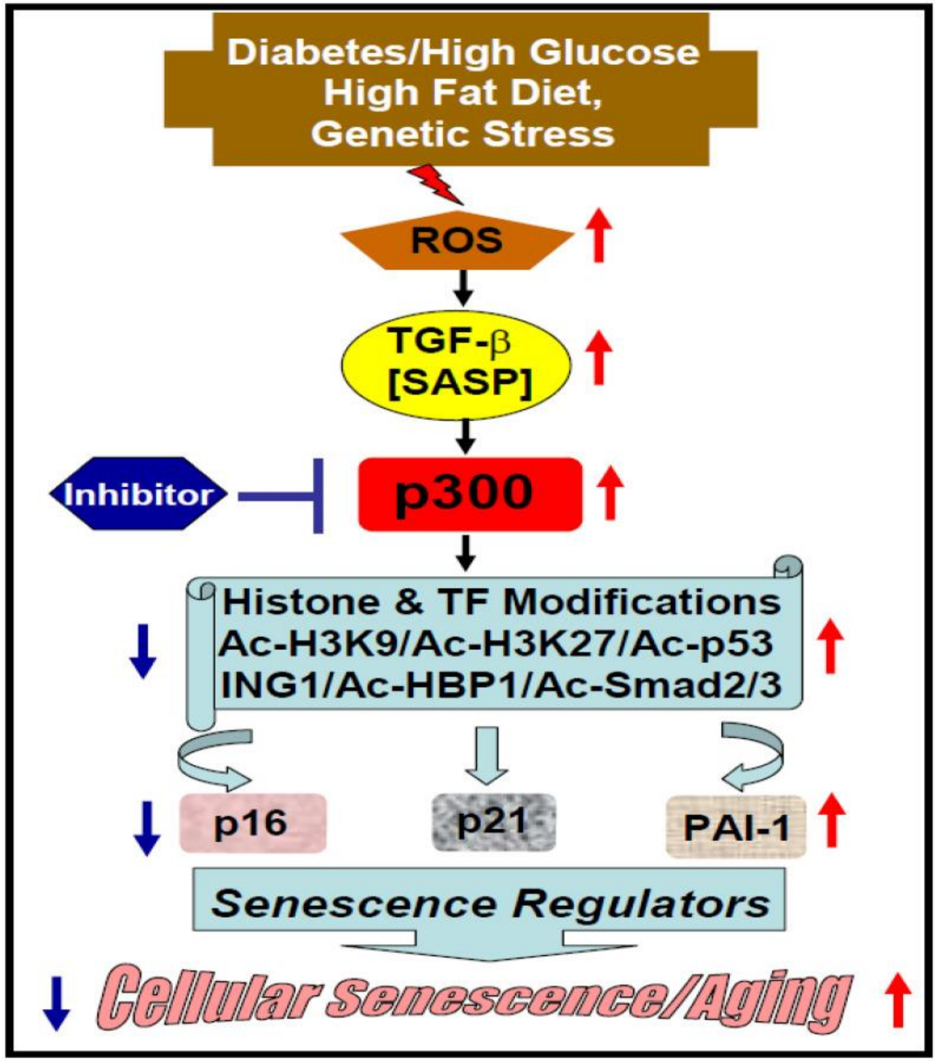

Figure: Model depicting the possible contribution of acetyltransferase p300 in cellular senescence/aging. ROS: Reactive Oxygen Species, TGF-β: Transforming Growth Factor-β. TF: Transcription factor, SASP: Senescence-Associated Secretory Phenotype, Ac: Acetylated histone/TF, ING1: Inhibitor of Growth 1 (Ghosh, 2021).

CONTENTS

- Disease characteristics
- Target characteristics
- **Therapeutic rationale**
- Expression profile
- Human genetic phenotype
- Animal genetic phenotype
- Competitive landscape
- Medicinal chemistry tools
- Preclinical evidence
- Clinical evidence

P300 - ROLE IN FIBROSIS

- **Wnt/β-catenin** has been **associated with organ fibrosis**. Following activation by upstream signalling, Wnt/β-catenin translocates to the nucleus. **Nuclear β-catenin recruits CBP or p300, to stimulate the transcription of its target genes**. Distinct roles have been reported for CBP and p300 (Yao, 2018 and references therein).

- **Transforming growth factor (TGF-β) promotes fibrogenesis** mainly through the SMAD family of transcription factors. SMAD3 is an effective profibrogenic transcription factor, which has long been reported as a regulator of the signalling pathway downstream of TGF. **p300/CBP functions as the regulator of liver fibrosis through modulating SMAD3 activity** (Yao, 2018 and references therein). **TGF-β stimulates COL1A2 transcription via functional cooperation between Smad3 and p300/CBP transcriptional coactivators** (Inagaki, 2003).

- **Under hypoxic conditions**, HIF-1α accumulates and dimerizes with HIF-1β, forming a protein heterodimer that complexes with p300/CBP in the nucleus. The **HIF-1α-p300/CBP complex** then binds to the hypoxia response element (HRE) to **activate** the transcription of genes involved in **fibrogenesis** (Yao, 2018).

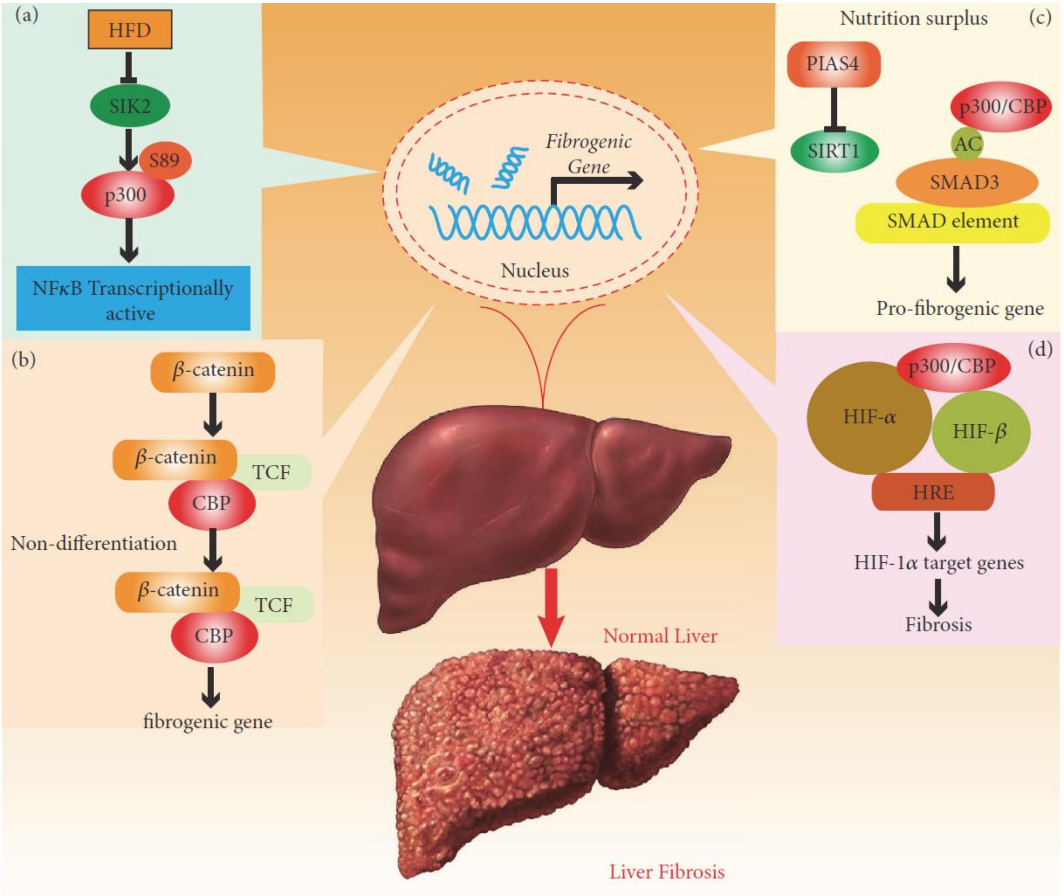

Figure: Potential contributions of p300/CBP to the development of proinflammatory response and liver fibrosis. (a) p300 histone acetyltransferases (HAT) activity inhibited by SIK2 activates phosphorylated NF-kB in HFD. (b) CBP/β-catenin-mediated transcription in fibrogenic genes expression. (c) p300/CBP acetylates K378 in the MH2 domain of SMAD3 and enhances the transcriptional activity of SMAD3. (d) p300/CBP interacts with HIF-1α forming the HIF-1α-p300/CBP complex and then binding to the hypoxia response element (HRE) to activate the transcription of genes involved in fibrogenesis. (SIK2: salt-inducible kinase 2; NF-κB: nuclear factor kappa B;HIF-1α: hypoxia-inducible factor-1alpha) (Yao, 2018 and references therein).

P300 – ROLE IN HEPATIC LIPID METABOLISM

- **p300/CBP affects lipid metabolism** in different tissues and cells. Although lipids are primary for maintaining organismal homeostasis, many diseases, such as, obesity, **nonalcoholic fatty liver disease**, and type 2 diabetes are associated with the **disordered lipid synthesis** (Yao, 2018).

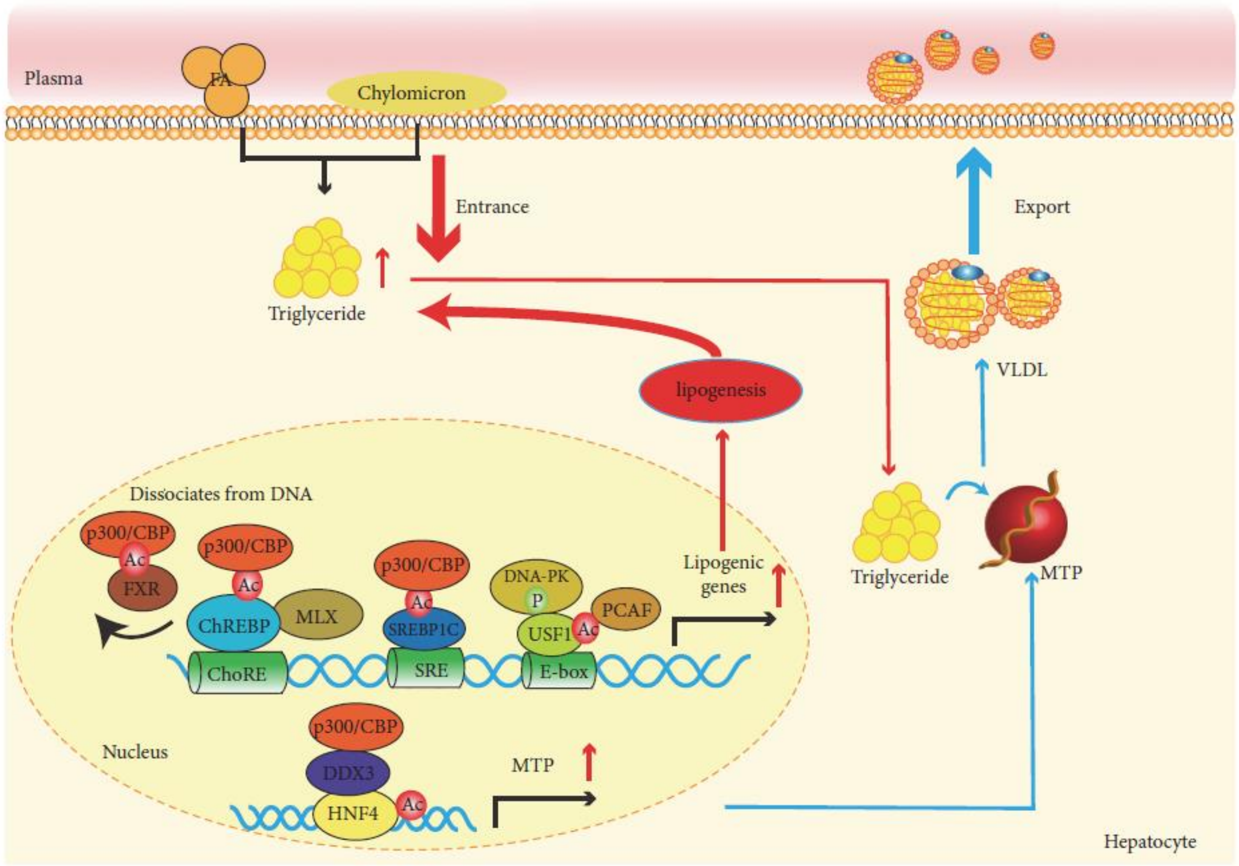

Figure: Potential contributions of p300/CBP to development of hepatic lipid metabolism. Red arrow: hepatic de novo lipogenesis and hyperlipidemia in p300/CBP regulation: the scheme indicates the regulatory actions of the inducible p300/CBP acetylation and their effects on de novo lipogenesis in both enzymatic and transcriptional events. Blue arrow: the regulation of p300/CBP in lipid export by interacting with DDX3 and inducing acetylation of HNF4 which increased the MTP promoter activation (HNF4: hepatocyte nuclear factor 4; MTP: microsomal triglyceride transfer protein) (Yao, 2018).

P300 – ROLE IN INFLAMMATION

- Activation of nuclear factor kappa B (NF-κB) induces inflammatory responses (Yao, 2018 and references therein). **Transcriptional activation of NF-κB requires association with p300/CBP** (Zhong, 2002).

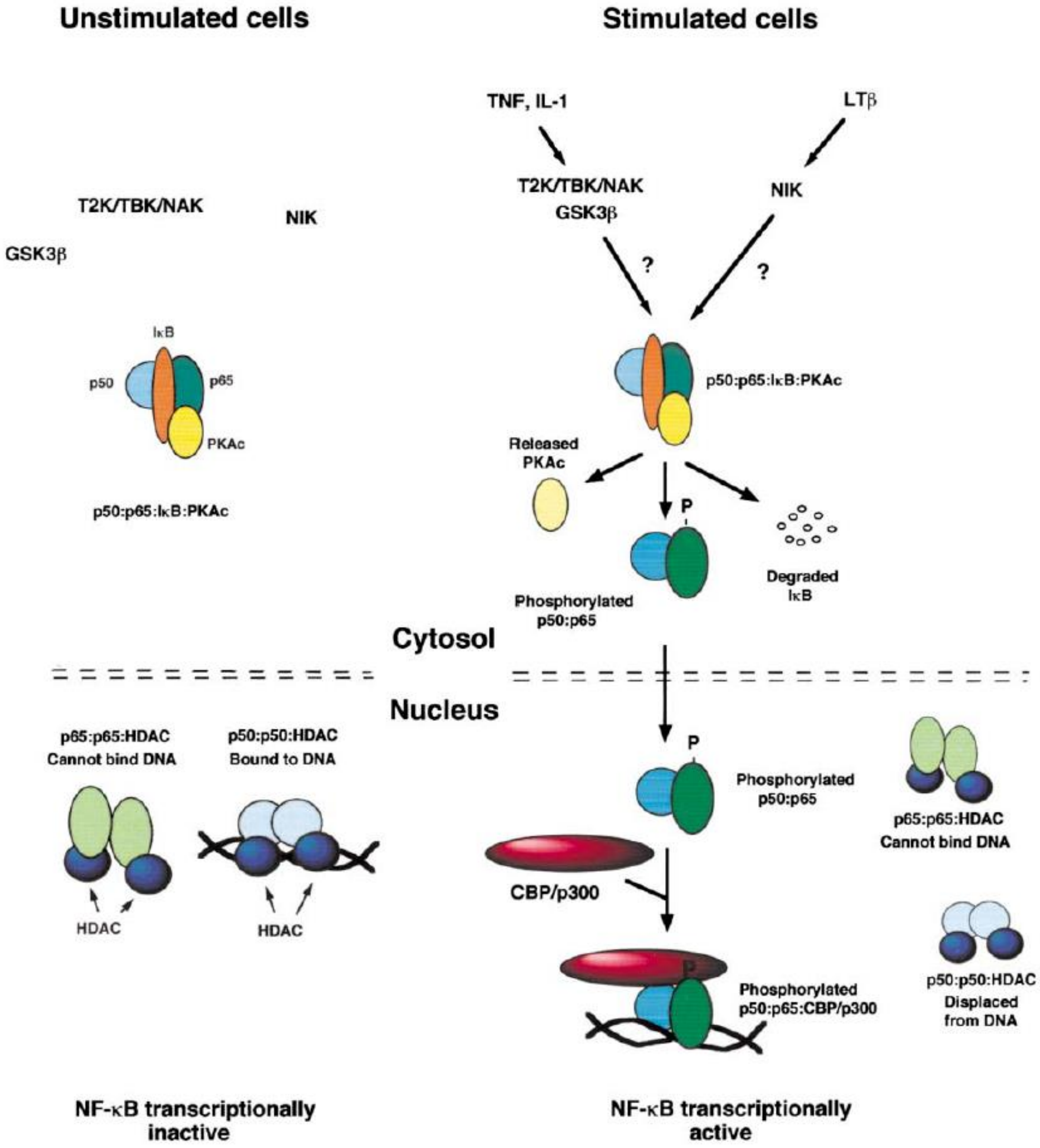

Figure: Phosphorylation Determines Whether Nuclear NF-κB Associates with Histone Acetylase or Deacetylase In unstimulated cells (left), transcriptionally inactive nuclear NF-κB consists of p50 or p65 homodimers bound to HDAC-1, and while p50- HDAC-1 binds to DNA, unphosphorylated p65-HDAC-1 complexes do not. In contrast, signal-induced transcriptionally active NF-κB entering the nucleus (right) is phosphorylated and associated with CBP/p300 and can displace p50-HDAC-1 complexes from DNA. This mechanism insures that only signal-induced NF-κB drives NF-κB-dependent gene expression (Zhong, 2002)

TABLE 1: Overview of p300/CBP regulates liver lipid, glucose metabolism, and liver fibrosis through regulating different targets.

Progress	Activity	Targets	Effect	Nutritional state	Metabolic response	Reference
Lipid metabolism	Acetylation	FXR	Inhibitory	High glucose and insulin	Lipogenesis↑ Gluconeogenesis↑	[17, 18]
	Acetylation	ChREBP	Stimulatory	High glucose and insulin	Lipogenesis↑	[19]
	Acetylation	SREBP1c	Stimulatory	High glucose and insulin	Lipogenesis↑	[20]
	Binding	DDX3	Stimulatory	/	Lipid export↑	[21]
Glucose metabolism	Acetylation	FOXO1	Stimulatory	Fasting state	Gluconeogenesis↑	[22]
	Binding	CREB	Stimulatory	Feeding state	Gluconeogenesis↑	[23]
Liver fibrosis	Binding	NF-κB	Stimulatory	High-fat diet	Proinflammatory response↑	[24]
	Binding	β-Catenin	Stimulatory	/	Liver fibrosis↑	[25]
	Acetylation	SMAD3	Stimulatory	High-fat high-carbohydrate	Liver fibrosis↑	[26]
	Binding	HIF-1α	Stimulatory	/	Liver fibrosis↑	[27]

(Yao, 2018).

CONTENTS

- Disease characteristics
- Target characteristics
- Therapeutic rationale
- **Expression profile**
- Human genetic phenotype
- Animal genetic phenotype
- Competitive landscape
- Medicinal chemistry tools
- Preclinical evidence
- Clinical evidence

TNO innovation for life

EXPRESSION PROFILE – RNA (HUMAN PROTEIN ATLAS)

RNA EXPRESSION OVERVIEW[i]

Consensus dataset[i]

RNA tissue specificity: Low tissue specificity

Organ | Expression | Alphabetical

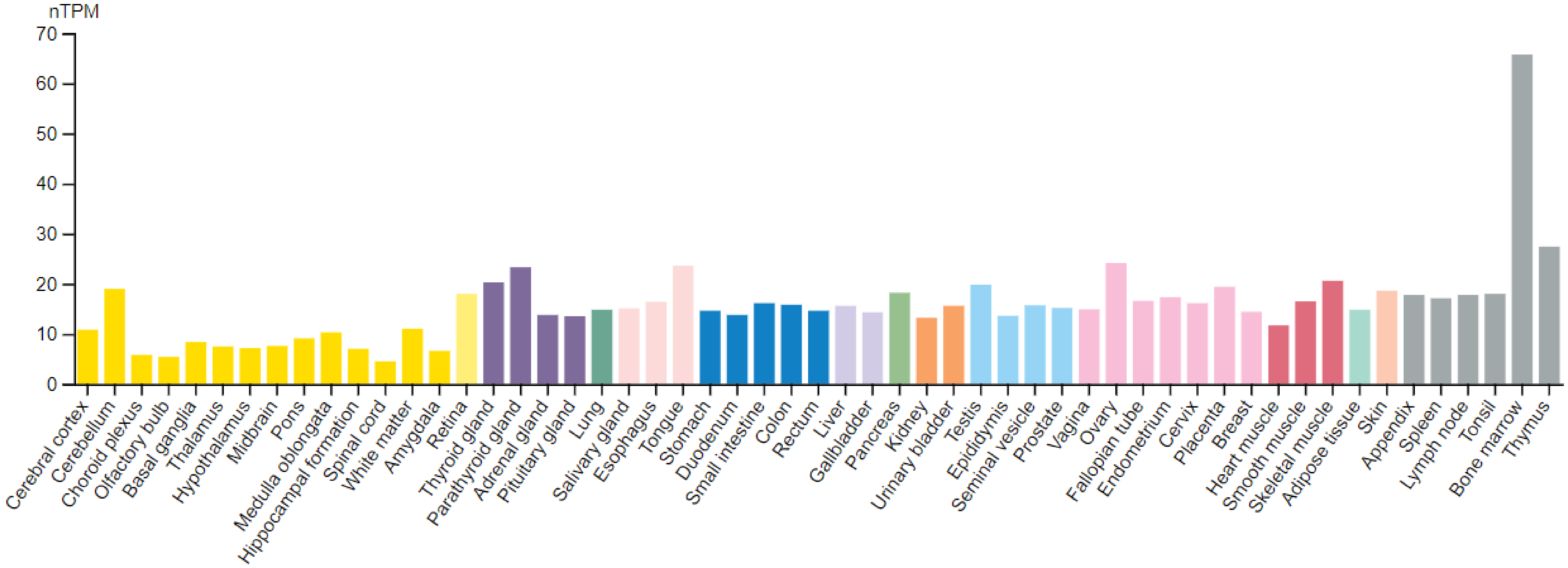

EXPRESSION PROFILE – PROTEIN (HUMAN PROTEIN ATLAS)

PROTEIN EXPRESSION OVERVIEW

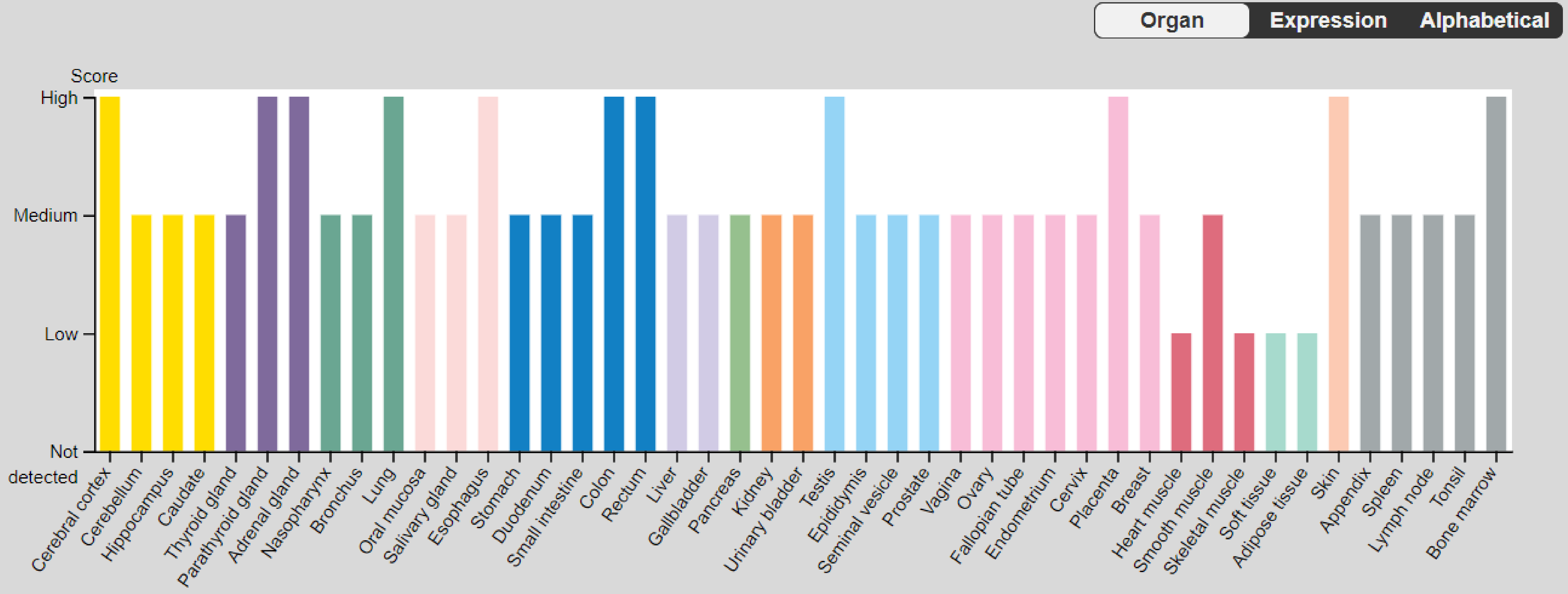

EXPRESSION PROFILE

- p300-C/EBPα/β pathway is activated in livers of patients with NAFLD (Jin, 2013).
- Expression of p300 was increased in livers of mice following injection of CCl_4 (Dou, 2018).

innovation for life

CONTENTS

- Disease characteristics
- Target characteristics
- Therapeutic rationale
- Expression profile
- **Human genetic phenotype**
- Animal genetic phenotype
- Competitive landscape
- Medicinal chemistry tools
- Preclinical evidence
- Clinical evidence

RELEVANCE FOR DISEASE – HUMAN GENETIC PHENOTYPE

- None.

CONTENTS

- Disease characteristics
- Target characteristics
- Therapeutic rationale
- Expression profile
- Human genetic phenotype
- **Animal genetic phenotype**
- Competitive landscape
- Medicinal chemistry tools
- Preclinical evidence
- Clinical evidence

ANIMAL GENETIC PHENOTYPE

- $P300^{F/Fcre}$ (conditional p300 KO mice) and control mice were given intraperitoneal injections of carbon tetrachloride (CCl_4) to induce liver fibrosis**. HSC activation and collagen deposition were reduced in livers of $p300^{F/Fcre}$ mice** compared with control mice (Dou, 2018).

- Livers of **transgenic mice expressing a dominant negative p300 molecule** (dnp300) were **resistant to CCl4-mediated injury** and showed reduced apoptosis but increased proliferation after injury (Breaux, 2015).

- During liver fibrosis, liver sinusoidal endothelial cells (LSECs) release angiocrine signals to recruit inflammatory cells into the liver. CCl4 or partial inferior vena cava ligation (pIVCL) were used to induce liver injury. Portal pressure and **liver fibrosis were reduced in mice with LSEC-specific p300 deletion ($p300^{LSEC\Delta/\Delta}$ mice) compared to $p300^{fl/fl}$ control mice following liver injury**. Accumulation of macrophages was also reduced in $p300^{LSEC\Delta/\Delta}$ mouse livers. Ccl2 was the most upregulated chemokine in injured LSECs but its increase was abrogated in $p300^{LSEC\Delta/\Delta\ mice}$. It has been suggested that **endothelial p300 interaction with NFκB and BRD4 increases C-C motif chemokine ligand 2 (CCL2) expression, leading to macrophage accumulation, portal hypertension and liver fibrosis**. **Inhibition of p300 and its binding partners might serve as novel therapies in the treatment of liver diseases** (Gao, 2021).

- Mice lacking the CH1 domain of p300 were resistant to high-fat-diet-mediated elevations of triglyceride levels, insulin resistance, and glucose intolerance (Breaux 2015 and references therein).

ANIMAL GENETIC PHENOTYPE

- The underlying mechanism of hepatic steatosis in old wild-type mice and in young S193D mice (mice with age-associated epigenetic changes, developing hepatic steatosis at 2 months of age) includes increased amounts of tripartite p300-C/EBPα/β complexes that activate promoters of five genes that drive triglyceride synthesis. **Knockdown of p300 in old wild-type mice inhibited hepatic steatosis. Transgenic mice expressing dominant-negative p300 had fewer C/EBPα/β-p300 complexes and did not develop age-dependent hepatic steatosis** (Jin, 2013).

CONTENTS

- Disease characteristics
- Target characteristics
- Therapeutic rationale
- Expression profile
- Human genetic phenotype
- Animal genetic phenotype
- **Competitive landscape**
- Medicinal chemistry tools
- Preclinical evidence
- Clinical evidence

COMPETITIVE LANDSCAPE

- Several small molecule inhibitors of p300 are in (pre)clinical development, almost all for the treatment of cancer, inlcuding HCC. One compound is in development for the treatment of IPF. Inobrodib (CCS-1477) is in the highest phase of development (phase I/II):

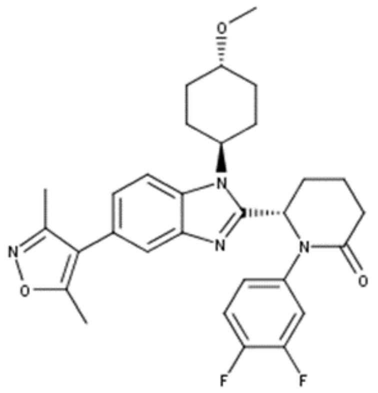

Microsoft Excel Worksheet

CONTENTS

- Disease characteristics
- Target characteristics
- Therapeutic rationale
- Expression profile
- Human genetic phenotype
- Animal genetic phenotype
- Competitive landscape
- **Medicinal chemistry tools**
- Preclinical evidence
- Clinical evidence

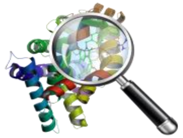

MEDICINAL CHEMISTRY TOOLS

- He (2021) has provided an overview of the various chemical classes of CBP/P300 inhibitors reported from 2010 to 2020.

- Several EP300 inhibitors are commercially available, e.g.:
  - ep300 | MedChemExpress Life Science Reagents
  - Selleck ep300 (selleckchem.com)

- siRNA/shRNA is also commercially available, e.g. https://www.thermofisher.com/; https://www.scbt.com/

- Functional assays described in literature (see respective sections):
  - Fibrotic gene expression
  - HSC activation

- Chemiluminescent and binding assays (FRET) for p300 inhibition are available/have been described, e.g:
  - P300 Chemiluminescent Assay Kit (bpsbioscience.com)
  - Early Drug-Discovery Efforts towards the Identification of EP300/CBP Histone Acetyltransferase (HAT) Inhibitors - Huhn - 2020 - ChemMedChem - Wiley Online Library

CONTENTS

- Disease characteristics
- Target characteristics
- Therapeutic rationale
- Expression profile
- Human genetic phenotype
- Animal genetic phenotype
- Competitive landscape
- Medicinal chemistry tools
- **Preclinical evidence**
- Clinical evidence

PRECLINICAL EVIDENCE – LIVER FIBROSIS

- Exposure to urocortin increased markedly the expression of collagen IV (Col4) in LX-2 cells (human HSC cell line). **Blocking p300 with garcinol efficiently suppressed the urocortin induced Col4 expression in LX-2 cells** and unveiled an apoptosis-inducing effect of urocortin (Wang, 2016)

- **Adefovir dipivoxil reduced HSC activation in vitro and fibrogenesis in mice administered CCL4**. Adefovir dipivoxil treatment **blocked CCl4-induced increase of p300 protein level** (Liu, 2019).

- **Nicotinamide riboside** (NR) **protected mice against liver fibrosis** induced by CCl4. NR suppressed activation of HSCs induced by TGF-β. NR protected mice against liver fibrosis via increasing the activity of Sirt1 and **decreasing the expression of P300**, resulting in the deacetylation of Smads in stellate cells (Jiang, 2019).

- TGF-β induced unfolded protein response (UPR) signalling in HSCs (LX-2), which acts in a feed-forward mechanism through IRE1α to promote fibrotic gene expression through C/EBPβ-p300. **p300 knockdown disrupted TGF-β or UPR-induced HSC activation, and pharmacological inhibition of the C/EBPβ–p300 complex decreased TGF-β -induced HSC activation** (Liu, 2019).

- p300 was found to facilitate TGF-β1-stimulated HSC activation by both noncanonical (cytoplasm-to-nucleus shuttle for SMAD2/3 and TAZ) and canonical (histone acetylation) mechanisms. TGF-β1 promoted binding of SMAD2/3 and TAZ to p300 and that p300 inactivation disrupted TGF-β1-mediated SMAD2/3 and TAZ nuclear accumulation. Deleting the p300 nuclear localization signal blocked TGF-β1-induced SMAD2/3 and TAZ nuclear transport. Consistently, p300 inactivation suppressed TGF-β1-mediated HSC activation (Wang, 2019).

PRECLINICAL EVIDENCE – LIVER FIBROSIS

- IFN-alpha, when administered into transgenic mice harboring the COL1A2 promoter sequence, significantly repressed promoter activation and prevented the progression of hepatic fibrosis induced by carbon tetrachloride injection. Transient transfection assays indicated that IFN-alpha decreased the steady-state levels of COL1A2 messenger RNA (mRNA) and inhibited basal and TGF-beta/Smad3-stimulated COL1A2 transcription in activated hepatic stellate cells (HSC). These **inhibitory effects of IFN-alpha on COL1A2 transcription were exerted through the interaction between phosphorylated Stat1 and p300** (Inagaki, 2003).

- The involvement of p300 in the inhibition of COL1A2 transcription by IFN-α has been evaluated in transfected CFSC-2G (activated rat hepatic stellate (HSC) clone established from a carbon tetrachloride (CCL4)-induced cirrhotic liver) and CF37 cells (primary cultures of human foetal skin fibroblasts). **Overexpression of the dominant negative p300 completely abolished the inhibitory effects of both IFN-α and IFN-γ on COL1A2 transcription in CFSC-2G cells as well as in CF37 fibroblasts** (Inagaki, 2003).

- Depletion of peroxisome proliferator-activated receptor γ **(PPARγ) represents one of the key molecular changes that underlie transdifferentiation (activation) of HSC in the genesis of liver fibrosis**. Ectopic expression of PPARγ suppressed HSC activation markers, most notably expression of α1(I) procollagen. In HSCs derived from normal rats, **PPARγ reduced NF-I-mediated α1(I) collagen promoter activity via its ability to inhibit p300-facilitated binding of NF-I to DNA** (Yavrom, 2005).

- In addition, the **competitive binding of phosphorylated Stat1 and Smad3 to a limited amount of p300/CBP has been implicated in mediating the antagonistic effects of IFN-γ on TGF-β-stimulated COL1A2 transcription** (Inagaki, 2003 and references therein)

PRECLINICAL EVIDENCE – LIVER FIBROSIS

- The activation of adenosine monophosphate-activated protein kinase (AMPK) modulates HSCs activation. Human (**LX-2**) and rat (**CFSC-2G**) HSC lines were **treated with TGF-b** to induce fibrogenic activation of HSCs. Pharmacological activation of AMPK by treatment with 5-aminoimidazole-4-carboxamide-1-beta-4-ribofuranoside (AICAR), metformin, or adiponectin lowered TGF-b-induced expression of **COL1A** and myofibroblast marker alpha-smooth muscle actin (**a-SMA**). Transient transduction of constitutively active AMPKa (caAMPKa) was sufficient to attenuate COL1A and a-SMA expression, whereas an AMPK inhibitor considerably abrogated the inhibitory effect of AICAR on fibrogenic gene expression. Although AMPK significantly suppressed Smad-dependent transcription, it did not affect TGF-b-stimulated phosphorylation, nuclear localization, or DNA-binding activity of Smad2/3. **AICAR rather attenuated TGF-b-induced Smad3 interaction with transcriptional coactivator p300 accompanying with reduction of Smad3 acetylation**. Moreover, **AICAR induced** not only physical interaction between AMPK and p300 but also **proteasomal degradation of p300 protein** (Lim, 2011).

PRECLINICAL EVIDENCE – NON-LIVER FIBROSIS (HEART)

- **P300** plays an important role in **cardiac fibrosis** (Ghosh, 2021):
  - **Phenylephrine (PE)-induced cardiac hypertrophy and myocardial fibrosis** in rodents is used as a widely-accepted animal model of hypertension induced cardiovascular complications in humans. Acetyltransferase **p300-induced acetylation of transcription factors including GATA4 plays a pivotal role in Phenylephrine (PE)-induced cardiac hypertrophy and heart failure**. Cdk9 was identified as an interacting factor with p300-GATA4 complex, and Cdk9 kinase activity is required for PE-induced phosphorylation of p300.
  - Pharmacological **inhibition of p300** acetyltransferase activity **with L002**, a small molecule inhibitor, **inhibits AngII-mediated** hypertension-induced ventricular wall thickness, cardiac hypertrophy, and **fibrosis** without mitigation of AngII-induced increased blood pressure. Mechanistically, p300 inhibitor **L002 ameliorates AngII downstream effector TGF-β-induced cardiac abnormalities** through inhibition of specific histone acetylation, myofibroblast differentiation, and matrix protein collagen synthesis in cardiac fibroblasts.
  - **p300 inhibitors L002 and C646 reverse** AngII-mediated hypertension-induced histone H3K9 acetylation, myofibroblast differentiation in myocardial tissues, left ventricular wall thickness, cardiac hypertrophy and **myocardial fibrosis in a murine model**.
  - Acetyltransferase p300 is involved in transverse aortic constriction (TAC)-mediated left ventricular pressure overload-induced cardiovascular pathologies.

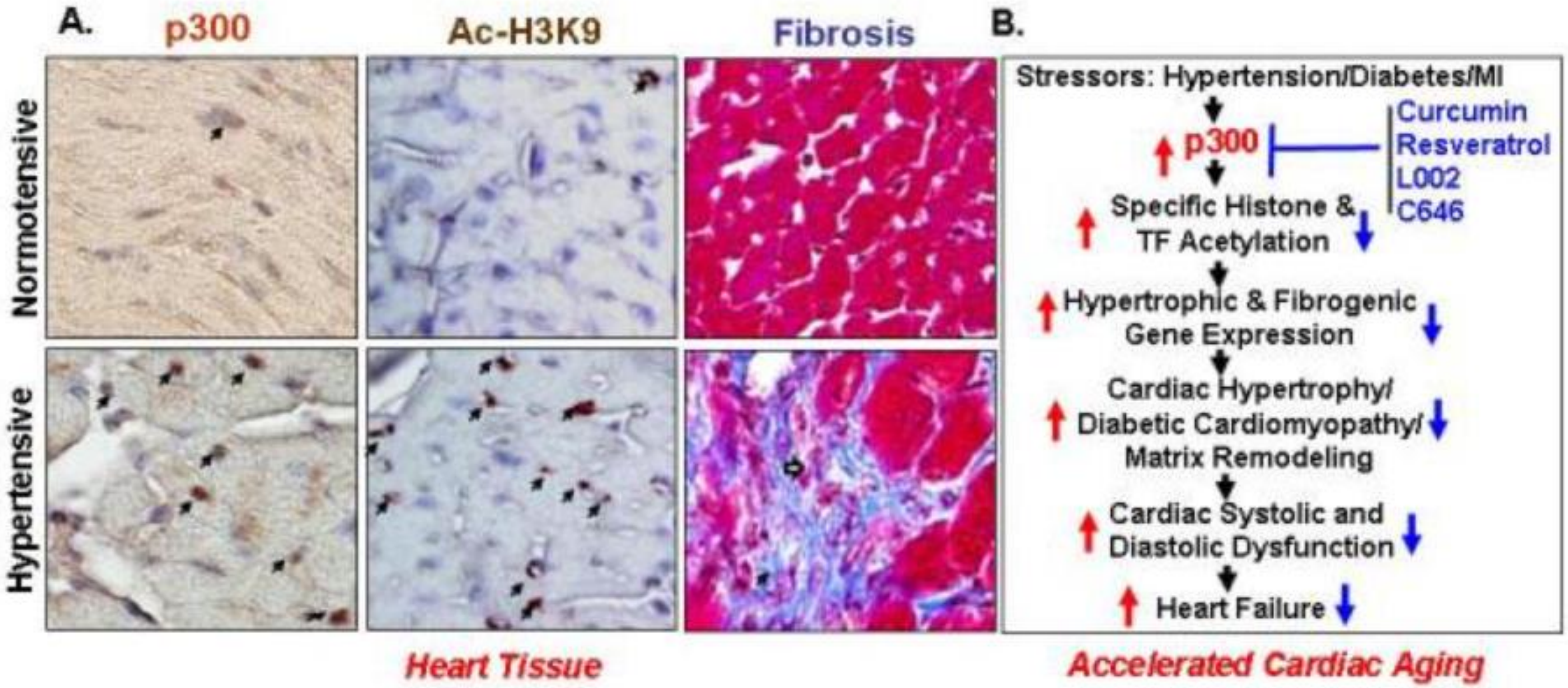

Figure: Acetyltransferase **p300** (p300), Acetylated histone 3 lysine 9 (AcH3K9) and **Type I collagen** (fibrosis) are elevated in hypertensive murine hearts. (**A**) Elevated expression of p300 in Angiotensin II-induced hypertensive murine hearts and its correlation with increased expression of acetylated histone H3 lysine 9 residue (AcH3K9) and elevated levels of ventricular fibrosis. (**B**) Model depicting the epigenetic regulation of accelerated cardiac aging by deregulated p300 and as a druggable target for accelerated cardiac aging therapy. TF; Transcription factor; MI: myocardial infarction. Inhibitors and modulators of p300 activity used in cell and animal models to suppress the acetyltransferase activity of p300 in hearts and accelerated cardiac aging pathologies: curcumin; L002; C646; resveratrol (Ghosh, 2020).

PRECLINICAL EVIDENCE – NON-LIVER FIBROSIS (LUNG)

- By exploiting the availability of samples from patients with Dupuytren's disease (DD) and a combination of epigenetic and transcriptional profiling studies, **EP300 was defined as an important regulator of human fibrosis** that directs diverse cellular processes in myofibroblasts. Collagen VI was found to be a downstream target of EP300 and is widely expressed in DD nodules and IPF lung tissue and mediates an important role in regulating ECM production, chemotaxis, and contractility (Williams, 2020).

Figure: Schematic illustrating the epigenetic control by EP300 of the profibrotic phenotype of myofibroblasts in Dupuytren's disease. Persistent activation of EP300 acetylates histones and transcription factors to promote extracellular matrix production and myofibroblast contractility. Collagen VI, a key target of EP300, plays a dominant role in regulating contraction. Proteolytic cleavage by PCSK7 generates small bioactive collagen VI fragments which control recruitment of immune cells by chemokine production, thereby perpetuating the cycle of chronic inflammation and fibrosis by the secretion of cytokines such as TNF (Williams, 2020).

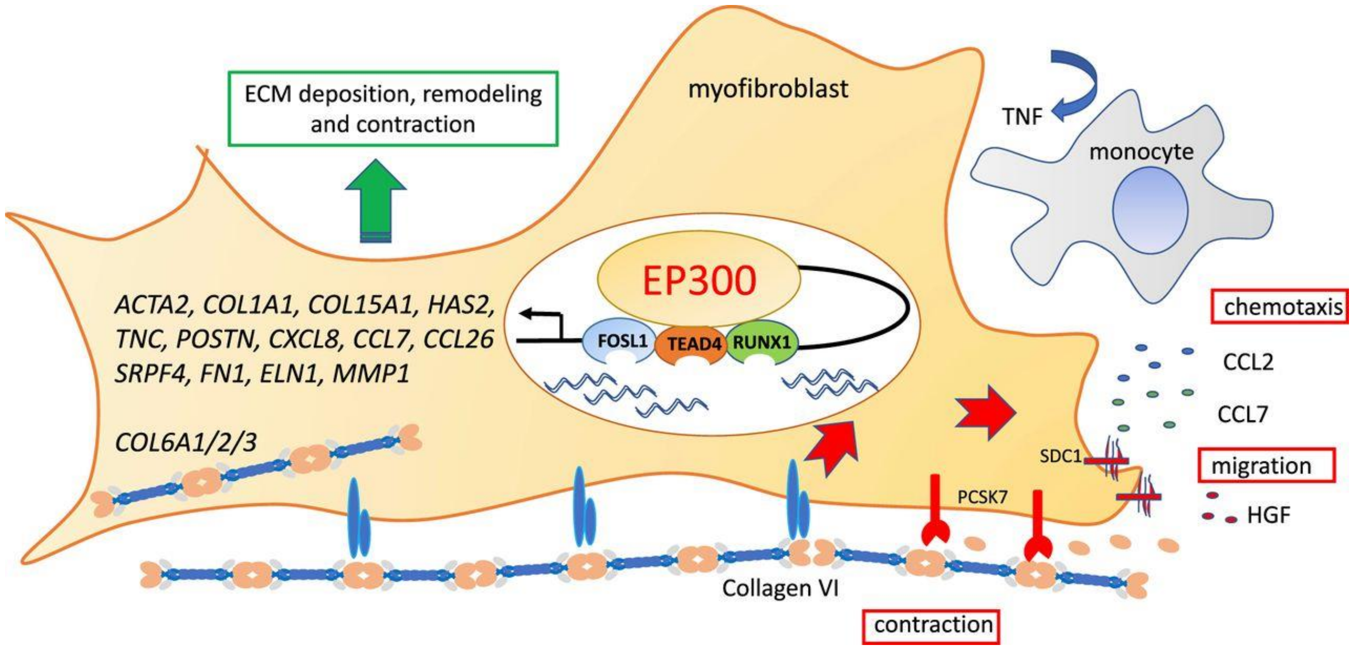

PRECLINICAL EVIDENCE – NON-LIVER FIBROSIS (LUNG)

- **EP300 inhibition reduced fibrotic hallmarks** in vitro using **primary fibroblasts from Ctrl and IPF patients**, in vivo using the **bleomycin mouse model**, and ex vivo using precision-cut lung slices (PCLS). The strongly suggest that inhibition of the bromodomain is crucial for the role of EP300 during IPF. *Whereas inactivation of cytosolic HDAC might be favorable for IPF patients, inactivation of nuclear HDAC might be counterproductive*. *Thus, a therapy against IPF based on EP300 inhibition might be more specific by targeting and reconstituting nuclear HDAC1 activity. Supporting this line of ideas, EP300 inhibition has been shown to ameliorate fibrosis in other organs* (Rubio, 2019).

- **TGF-β1 activated** FN1, **collagen**, and DDR1 signaling could be **reversed by the combination of p300 siRNA and DDR1 inhibitors**. Moreover, the EP300 inhibitor SGC-CBP30 displayed synergistic effects with DDR1 inhibitors in pathogenic scores, airway goblet cell counts in bronchoalveolar lavage fluid (BALF), IL-4, IFN-γ, FN1COL1 A1 secretion and α-SMA, a marker of myofibroblast (Tao, 2018).

PRECLINICAL EVIDENCE – NON-LIVER FIBROSIS (SKIN)

- **IFN-g abrogates** TGF-b-stimulated **COL1A2 transcription** in fibroblasts by inhibiting Smad activities. IFN-g appears **to induce competition between activated Stat1a and Smad3 for interaction with limiting amounts of cellular p300/CBP**. Overexpression of p300 restored COL1A2 stimulation by TGF-b in the presence of IFN-g, and potentiated IFN-g-dependent positive transcriptional responses. In contrast to fibroblasts, in U4A cells lacking Jak1 and consequently unable to activate Stat1a-mediated responses, IFN-g failed to repress TGF-b-induced transcription. These results indicate that as essential coactivators for both Smad3 and Stat1a, nuclear p300/CBP integrate signals that positively or negatively regulate COL1A2 transcription (Gosh, 2001).

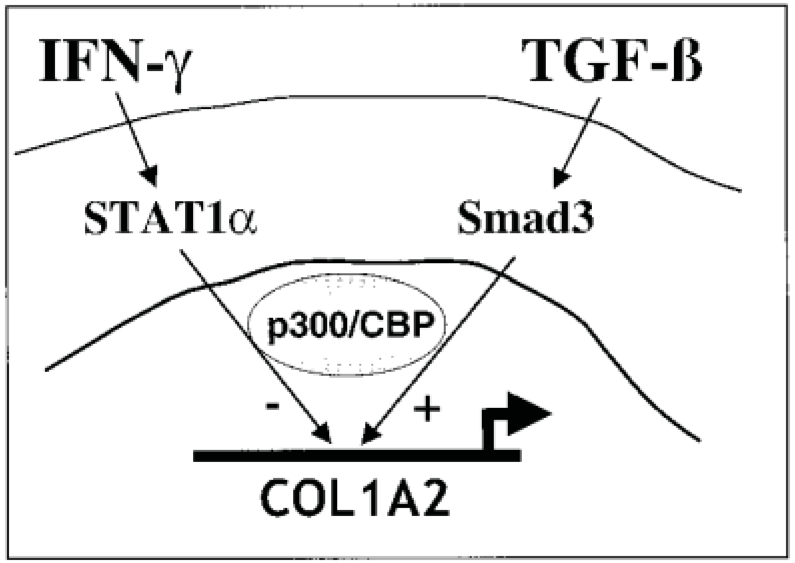

Figure: Antagonistic regulation of COL1A2 transcription in primary fibroblasts by TGF-b (1) and IFN-g (2). The two cytokines exert opposing effects on gene expression through competition between Smads and STAT1 for limiting amounts of the shared cellular coactivators p300/CBP (Gosh, 2001).

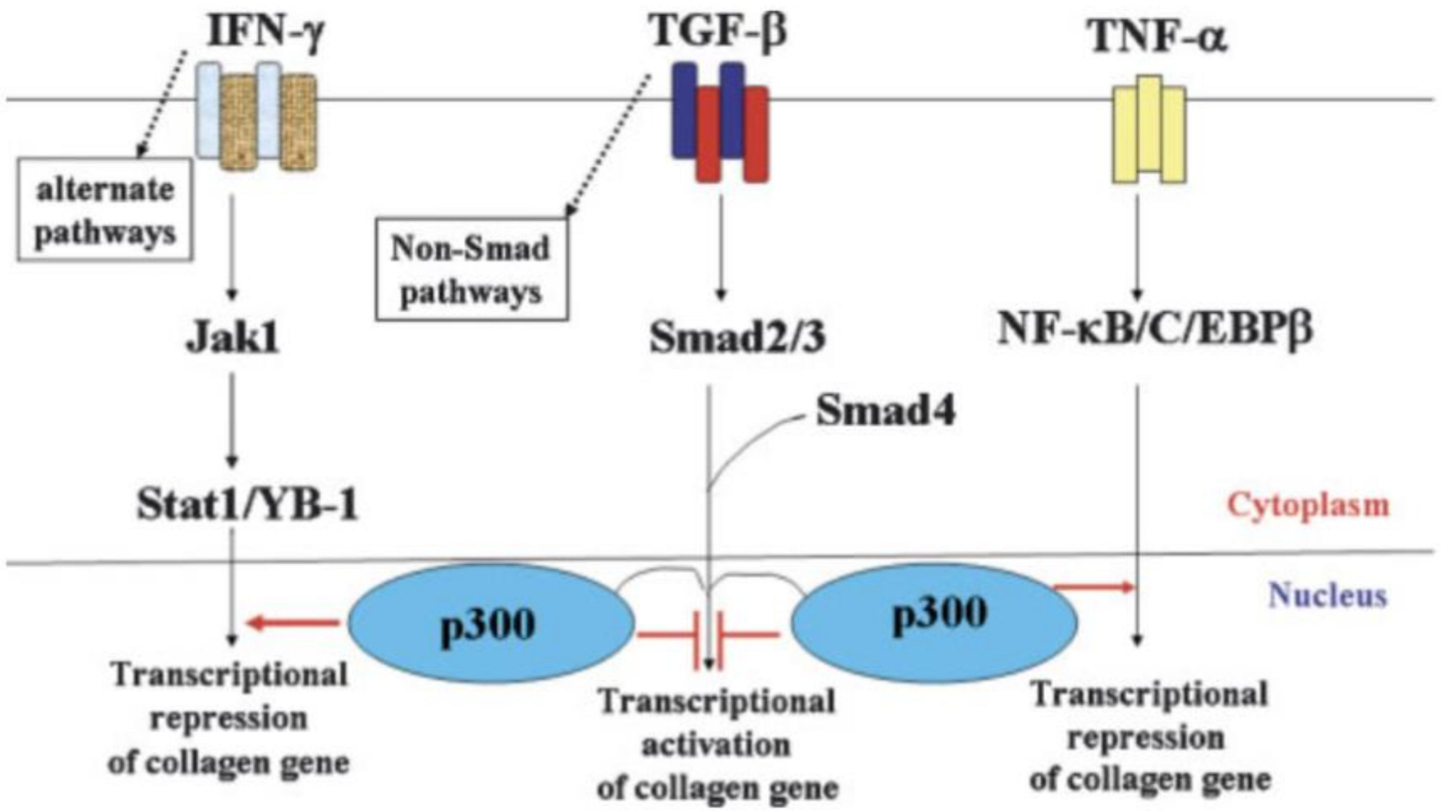

Figure: Regulation of collagen synthesis by extracellular signals: convergence on p300. Three major cytokines and corresponding signal transduction pathways controlling collagen gene expression and cross talks among these pathways are shown (Ghosh, 2007).

PRECLINICAL EVIDENCE – LIPID METABOLISM / NAFLD

- 3,4-dihydroxyphenolacetic acid (DHPAA), 3,4-hydroxyphenylacetic acid (HPAA) and 3,4-dihydroxytoluene (DHT), significantly inhibited in vitro HAT activity with DHT having the strongest inhibitory activity. **DHT was shown to be a highly efficient inhibitor of p300 HAT activity**, which corresponded with its high degree of **inhibition on intracellular lipid accumulation in HepG2 cells**. **In HepG2 cells, DHT concentration dependently abrogated p300-histone binding and induced hypoacetylation of histone subunits H3K9, H3K36, H4K8 and H4K16, eventually leading to the downregulation of lipogenesis-related genes and attenuating lipid accumulation**. In **ob/ob mice, administration of DHT dose-dependently improved the NAFLD pathogenic features** including body weight, liver mass, fat mass, lipid accumulation in the liver, and biochemical blood parameters, accompanied by the decreased mRNA expression of lipogenic genes in the liver. It has been suggested that **DHT, a novel p300 histone acetyltransferase inhibitor, may be a potential preventive or therapeutic agent for NAFLD** (Lee, 2020).

- p300 forms complexes with C/EBP proteins and **activates promoters of genes involved in triglyceride synthesis during the development of hepatic steatosis**. Inhibition of histone acetyltransferase (HAT) activity of p300 decreased the incidence of hepatic steatosis (Breaux 2015 and references therein).

- **Nuclear factor erythroid-2-related factor-2 (Nrf2) activation inhibits liver X receptor-α (LXRα) activity and LXRα-dependent liver steatosis by competing with FXR for p300**, causing farnesoid X receptor (FXR) activation and FXR-mediated small heterodimer partner (SHP) induction (Kay, 2011).

- C57BL/6 mice fed with either normal chow diet (NCD) or high-fat diet (HFD) were administrated with **A-485 (a selective inhibitor of CBP/p300 histone acetyltransferase (HAT) activity),** for 1 week. The **white adipose tissue (WAT) weight and adipocyte size were reduced** in **A-485-administrated mice, with decreased expressions of lipogenic genes and transcriptional factors** (Zhou, 2020).

PRECLINICAL EVIDENCE – LIPID METABOLISM / NAFLD

- Following oleic-palmitic acid (OPA)-induced lipid accumulation in HepG2 cells, the acetylation status of histone and non-histone proteins, HAT activity, and mRNA expression of representative lipogenic genes, including PPARγ, SREBP-1c, ACLY, and FASN, were evaluated. Non-histone protein acetylation increased following OPA treatment and the acetylation of histones H3K9, H4K8, and H4K16 was accelerated, accompanied by an increase in HAT activity. **OPA-induced increases in the mRNA expression of lipogenic genes were down-regulated by C-646, a p300/CBP-specific inhibitor**. Finally, a positive correlation between HAT activity and lipid accumulation was detected (Chung, 2019).

PRECLINICAL INFORMATION – INFLAMMATION

- Salt-inducible kinase 2 (SIK2) can suppress p300 function through the inhibition of its histone acetyltransferases (HAT) activity via direct phosphorylation at Ser89. SIK2 reduced expression led to an inflammation characterized by increased secretion of IL-6 and TNF-α and stimulation of NF-κB activity via downregulation of p300 HAT activity in mice on high-fat diet (Yao, 2018 and references therein).

CONTENTS

- Disease characteristics
- Target characteristics
- Therapeutic rationale
- Expression profile
- Human genetic phenotype
- Animal genetic phenotype
- Competitive landscape
- Medicinal chemistry tools
- Preclinical evidence
- **Clinical evidence**

CLINICAL EVIDENCE

- None.

REFERENCES

- Breaux M, Lewis K, Valanejad L, Iakova P, Chen F, Mo Q, Medrano E, Timchenko L, Timchenko N. p300 Regulates Liver Functions by Controlling p53 and C/EBP Family Proteins through Multiple Signaling Pathways. Mol Cell Biol. 2015 Sep 1;35(17):3005-16. doi: 10.1128/MCB.00421-15. Epub 2015 Jun 22. PMID: 26100016; PMCID: PMC4525310.
- Dou C, Liu Z, Tu K, Zhang H, Chen C, Yaqoob U, Wang Y, Wen J, van Deursen J, Sicard D, Tschumperlin D, Zou H, Huang WC, Urrutia R, Shah VH, Kang N. P300 Acetyltransferase Mediates Stiffness-Induced Activation of Hepatic Stellate Cells Into Tumor-Promoting Myofibroblasts. Gastroenterology. 2018 Jun;154(8):2209-2221.e14. doi: 10.1053/j.gastro.2018.02.015. Epub 2018 Feb 15. PMID: 29454793; PMCID: PMC6039101.
- Chan HM, La Thangue NB. p300/CBP proteins: HATs for transcriptional bridges and scaffolds. J Cell Sci. 2001 Jul;114(Pt 13):2363-73. doi: 10.1242/jcs.114.13.2363. PMID: 11559745.
- Chung S, Hwang JT, Park JH, Choi HK. Free fatty acid-induced histone acetyltransferase activity accelerates lipid accumulation in HepG2 cells. Nutr Res Pract. 2019 Jun;13(3):196-204. doi: 10.4162/nrp.2019.13.3.196. Epub 2019 Apr 12. PMID: 31214287; PMCID: PMC6548710.
- Gao J, Wei B, Liu M, Hirsova P, Sehrawat TS, Cao S, Hu X, Xue F, Yaqoob U, Kang N, Cui H, Pomerantz WCK, Kostallari E, Shah VH. Endothelial p300 Promotes Portal Hypertension and Hepatic Fibrosis Through C-C Motif Chemokine Ligand 2-Mediated Angiocrine Signaling. Hepatology. 2021 Jun;73(6):2468-2483. doi: 10.1002/hep.31617. Epub 2021 Apr 19. PMID: 33159815; PMCID: PMC8102654.
- Ghosh AK, Yuan W, Mori Y, Chen Sj, Varga J. Antagonistic regulation of type I collagen gene expression by interferon-gamma and transforming growth factor-beta. Integration at the level of p300/CBP transcriptional coactivators. J Biol Chem. 2001 Apr 6;276(14):11041-8. doi: 10.1074/jbc.M004709200. Epub 2000 Dec 29. PMID: 11134049.
- Ghosh AK, Varga J. The transcriptional coactivator and acetyltransferase p300 in fibroblast biology and fibrosis. J Cell Physiol. 2007 Dec;213(3):663-71. doi: 10.1002/jcp.21162. PMID: 17559085.
- Ghosh AK. p300 in Cardiac Development and Accelerated Cardiac Aging. Aging Dis. 2020 Jul 23;11(4):916-926. doi: 10.14336/AD.2020.0401. PMID: 32765954; PMCID: PMC7390535.
- Ghosh AK. Acetyltransferase p300 Is a Putative Epidrug Target for Amelioration of Cellular Aging-Related Cardiovascular Disease. Cells. 2021 Oct 22;10(11):2839. doi: 10.3390/cells10112839. PMID: 34831061; PMCID: PMC8616404.
- He ZX, Wei BF, Zhang X, Gong YP, Ma LY, Zhao W. Current development of CBP/p300 inhibitors in the last decade. Eur J Med Chem. 2021 Jan 1;209:112861. doi: 10.1016/j.ejmech.2020.112861. Epub 2020 Oct 1. PMID: 33045661.

REFERENCES

- Inagaki Y, Nemoto T, Kushida M, Sheng Y, Higashi K, Ikeda K, Kawada N, Shirasaki F, Takehara K, Sugiyama K, Fujii M, Yamauchi H, Nakao A, de Crombrugghe B, Watanabe T, Okazaki I. Interferon alfa down-regulates collagen gene transcription and suppresses experimental hepatic fibrosis in mice. Hepatology. 2003 Oct;38(4):890-9. doi: 10.1053/jhep.2003.50408. PMID: 14512876.
- Jiang R, Zhou Y, Wang S, Pang N, Huang Y, Ye M, Wan T, Qiu Y, Pei L, Jiang X, Huang Y, Yang H, Ling W, Li X, Zhang Z, Yang L. Nicotinamide riboside protects against liver fibrosis induced by CCl_4 via regulating the acetylation of Smads signaling pathway. Life Sci. 2019 May 15;225:20-28. doi: 10.1016/j.lfs.2019.03.064. Epub 2019 Mar 27. PMID: 30928408.
- Jin J, Iakova P, Breaux M, Sullivan E, Jawanmardi N, Chen D, Jiang Y, Medrano EM, Timchenko NA. Increased expression of enzymes of triglyceride synthesis is essential for the development of hepatic steatosis. Cell Rep. 2013 Mar 28;3(3):831-43. doi: 10.1016/j.celrep.2013.02.009. Epub 2013 Mar 14. PMID: 23499441; PMCID: PMC3615099.
- Kay HY, Kim WD, Hwang SJ, Choi HS, Gilroy RK, Wan YJ, Kim SG. Nrf2 inhibits LXRα-dependent hepatic lipogenesis by competing with FXR for acetylase binding. Antioxid Redox Signal. 2011 Oct 15;15(8):2135-46. doi: 10.1089/ars.2010.3834. Epub 2011 Jun 13. PMID: 21504366; PMCID: PMC6468953.
- Lee J, Song JH, Chung MY, Lee JH, Nam TG, Park JH, Hwang JT, Choi HK. 3,4-dihydroxytoluene, a metabolite of rutin, suppresses the progression of nonalcoholic fatty liver disease in mice by inhibiting p300 histone acetyltransferase activity. Acta Pharmacol Sin. 2021 Sep;42(9):1449-1460. doi: 10.1038/s41401-020-00571-7. Epub 2020 Dec 10. PMID: 33303988; PMCID: PMC8379200.
- Lim JY, Oh MA, Kim WH, Sohn HY, Park SI. AMP-activated protein kinase inhibits TGF-β-induced fibrogenic responses of hepatic stellate cells by targeting transcriptional coactivator p300. J Cell Physiol. 2012 Mar;227(3):1081-9. doi: 10.1002/jcp.22824. PMID: 21567395.
- Liu Z, Li C, Kang N, Malhi H, Shah VH, Maiers JL. Transforming growth factor β (TGFβ) cross-talk with the unfolded protein response is critical for hepatic stellate cell activation. J Biol Chem. 2019 Mar 1;294(9):3137-3151. doi: 10.1074/jbc.RA118.005761. Epub 2019 Jan 4. PMID: 30610118; PMCID: PMC6398135.
- Rubio K, Singh I, Dobersch S, Sarvari P, Günther S, Cordero J, Mehta A, Wujak L, Cabrera-Fuentes H, Chao CM, Braubach P, Bellusci S, Seeger W, Günther A, Preissner KT, Wygrecka M, Savai R, Papy-Garcia D, Dobreva G, Heikenwalder M, Savai-Pullamsetti S, Braun T, Barreto G. Inactivation of nuclear histone deacetylases by EP300 disrupts the MiCEE complex in idiopathic pulmonary fibrosis. Nat Commun. 2019 May 20;10(1):2229. doi: 10.1038/s41467-019-10066-7. PMID: 31110176; PMCID: PMC6527704.

REFERENCES

- Tao J, Zhang M, Wen Z, Wang B, Zhang L, Ou Y, Tang X, Yu X, Jiang Q. Inhibition of EP300 and DDR1 synergistically alleviates pulmonary fibrosis in vitro and in vivo. Biomed Pharmacother. 2018 Oct;106:1727-1733. doi: 10.1016/j.biopha.2018.07.132. Epub 2018 Jul 30. PMID: 30119248.
- Williams LM, McCann FE, Cabrita MA, Layton T, Cribbs A, Knezevic B, Fang H, Knight J, Zhang M, Fischer R, Bonham S, Steenbeek LM, Yang N, Sood M, Bainbridge C, Warwick D, Harry L, Davidson D, Xie W, Sundström M, Feldmann M, Nanchahal J. Identifying collagen VI as a target of fibrotic diseases regulated by CREBBP/EP300. Proc Natl Acad Sci U S A. 2020 Aug 25;117(34):20753-20763. doi: 10.1073/pnas.2004281117. Epub 2020 Aug 5. PMID: 32759223; PMCID: PMC7456151.
- Wang C, Yang S, Huang J, Chen S, Li Y, Li Q. Activation of corticotropin releasing factor receptors up regulates collagen production by hepatic stellate cells via promoting p300 expression. Biol Chem. 2016 May;397(5):437-44. doi: 10.1515/hsz-2015-0233. PMID: 26756093.
- Wang Y, Tu K, Liu D, Guo L, Chen Y, Li Q, Maiers JL, Liu Z, Shah VH, Dou C, Tschumperlin D, Voneschen L, Yang R, Kang N. p300 Acetyltransferase Is a Cytoplasm-to-Nucleus Shuttle for SMAD2/3 and TAZ Nuclear Transport in Transforming Growth Factor β-Stimulated Hepatic Stellate Cells. Hepatology. 2019 Oct;70(4):1409-1423. doi: 10.1002/hep.30668. Epub 2019 May 23. PMID: 31004519; PMCID: PMC6783326.
- Yao W, Wang T, Huang F. p300/CBP as a Key Nutritional Sensor for Hepatic Energy Homeostasis and Liver Fibrosis. Biomed Res Int. 2018 May 15;2018:8168791. doi: 10.1155/2018/8168791. PMID: 29862292; PMCID: PMC59.
- Yavrom S, Chen L, Xiong S, Wang J, Rippe RA, Tsukamoto H. Peroxisome proliferator-activated receptor gamma suppresses proximal alpha1(I) collagen promoter via inhibition of p300-facilitated NF-I binding to DNA in hepatic stellate cells. J Biol Chem. 2005 Dec 9;280(49):40650-9. doi: 10.1074/jbc.M510094200. Epub 2005 Oct 10. PMID: 16216869.
- Zhong H, May MJ, Jimi E, Ghosh S. The phosphorylation status of nuclear NF-kappa B determines its association with CBP/p300 or HDAC-1. Mol Cell. 2002 Mar;9(3):625-36. doi: 10.1016/s1097-2765(02)00477-x. PMID: 11931769.
- Zhou F, Liu Q, Zhang L, Zhu Q, Wang S, Zhu K, Deng R, Liu Y, Yuan G, Wang X, Zhou L. Selective inhibition of CBP/p300 HAT by A-485 results in suppression of lipogenesis and hepatic gluconeogenesis. Cell Death Dis. 2020 Sep 11;11(9):745. doi: 10.1038/s41419-020-02960-6. PMID: 32917859; PMCID: PMC

BACKUP SLIDES
