# Supplementary material for: Integrating text mining with network models for successful target identification: in vitro validation in MASH-induced liver fibrosis
Source: Front Pharmacol. 2024 Sep 27;15:1442752. doi: 10.3389/fphar.2024.1442752 (PMC11466758; doi:10.3389/fphar.2024.1442752)
Supplement: Supplementary file 2 [file DataSheet1.PDF]

Supplementary Material 1

1 Supplementary Material

1.1 **Suppl 1.1** Fibrosis clusters used in our text-mining approach to associate genes/proteins with liver fibrosis.

**Cluster**	**Example term**
Hepatic stellate cell cluster	HSC activation
Extracellular matrix cluster	extracellular matrix crosslinking
Hepatocyte cluster	Hepatocyte apoptosis
Fibrosis-terms	Hepatic fibrosis
Collagen cluster	Collagen deposits
Cirrhosis cluster	Cirrhotic
Inflammation cluster	Hepatic inflammation
Cholangiocyte cluster	Cholangiocyte apoptosis
Fibrosis protein cluster	TGF-β/Smad
Fibrosis diagnosis	FIB-4 index
Fibroblast cluster	Myofibroblast activation
Kupffer cell cluster	Kupffer cell activation
Fibril cluster	Fibril formation
Non-liver fibrosis cluster	Cardiac fibrosis

1.2 Suppl. 1.2 PPI network

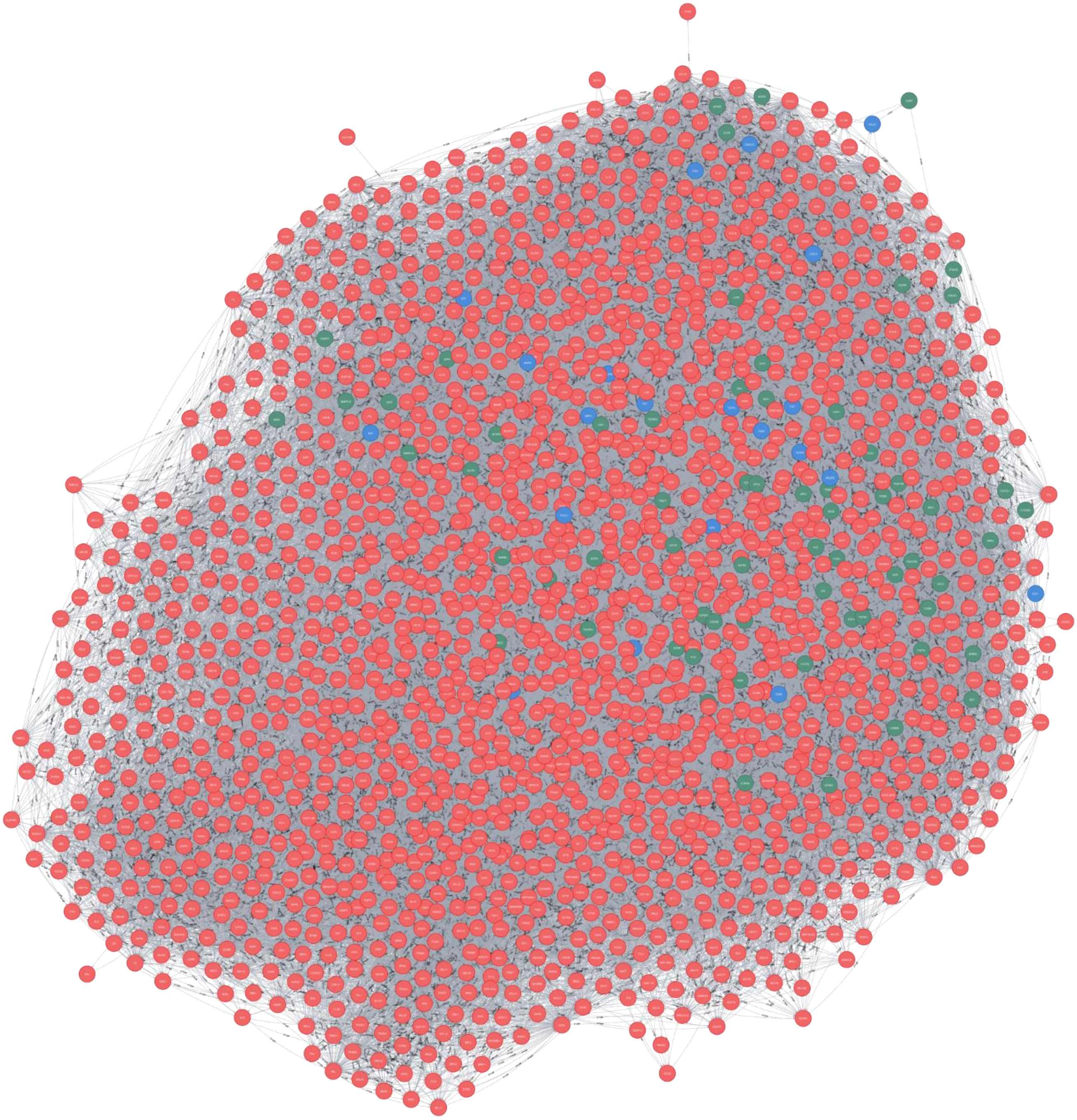

Figure 1.2 Constructed PPI disease network for MASH-related liver fibrosis. Blue = GFs; Green = CellTalkDB receptors; red = other nodes.

1.3 Suppl. 1.3 Criteria used for a quick scan analysis of candidate targets using database-derived information. The fibrosis count reflects the number of times the protein has been associated with a fibrotic feature in PubMed abstracts (see body text). An asterisk denotes that this information was also established for neighbours of the protein and included in the assessment of the target.

**Protein characteristics**	**Data Source**	**Link**
Function	UniProt	https://www.uniprot.org/
Fibrosis count*	TargetTri (PubMed text-mining)	https://www.targettri.com
Fibrosis neighbours	StringDB	https://string-db.org/
Disease pathways*	Comparative toxicogenomics database	https://ctdbase.org/
Human genetics*	OMIM	https://www.omim.org/
Murine genetics*	Mouse Genome Informatics	https://www.informatics.jax.org/
Expression	Human Protein Atlas	https://www.proteinatlas.org/
Ligands	Cortellis	https://cortellis.com/drugdiscovery
